# Soil environment, physiological metabolism and ornamental traits of Zinnia response to water and nitrogen combination in a cold and arid environment

**DOI:** 10.3389/fpls.2026.1785249

**Published:** 2026-03-31

**Authors:** Zhen Xu, Hengjia Zhang, Xietian Chen, Chenli Zhou

**Affiliations:** 1 College of Agriculture and Biology, Liaocheng University, Liaocheng, China; 2 Yimin Irrigation Experimental Station, Hongshui River Management Office, Zhangye, China

**Keywords:** comprehensive evaluation, photosynthetic rate, ornamental value, soil nutrients, water and nitrogen regulation

## Abstract

In response to the prominent issues of water shortage and intensified soil desertification in the arid regions of Northwest China, this study aimed to optimize the water and nitrogen (N) management plan for ornamental Zinnia. Using the tall Zinnia variety “Pink Dream” as the experimental material, a two-year field experiment was conducted in the oasis area of Hexi Corridor using the integrated water and fertilizer management technology under subsurface drip irrigation. Three irrigation gradients (W1, 55%∼65% field capacity (FC); W2, 65%∼75% FC; W3, 75%∼85% FC) and three N application levels (N1, 90 kg·ha^-1^; N2, 150 kg·ha^-1^; N3, 210 kg·ha^-1^) were respectively established. The local water and fertilizer management practice (W3 level combined with 270 kg·ha^-1^ N application) was used as the control (CK), resulting in a total of ten treatments. The effects of different water-N regulations on rhizosphere soil environment, plant physiological metabolism, growth and development, and ornamental traits of Zinnia were systematically explored. The results indicated that in both growing seasons, the W2N2 and W2N3 treatments significantly (*P* < 0.05) outperformed the CK and other treatments in terms of dry matter accumulation and ornamental value, with increases ranging from 7.20% to 153.47%. However, the optimal photosynthetic assimilation capacity and physiological and biochemical indicators were recorded in W2N2 treatment, which was significantly higher than that in CK and other treatments, with an increase of 8.76% to 45.50%. The W1N1 water and nitrogen management mode reduced Zinnia nutrient deficiency, decreased leaf area, photosynthetic rate, growth and physiological-biochemical indicators Also, the W3N3 mode did not enhance the above indicators while reduced its ornamental value. In addition, increasing the nitrogen fertilizer application rate from N1 to N3 led to an average increase of 14.51% and 37.56% in the total nitrogen (TN) and soil organic matter (SOM) content of the 0–60 cm soil layer, respectively. In contrast, raising the irrigation level from W1 to W3 resulted in an increase of 7.10% and 26.92% in the TN and SOM content, respectively. Entropy weight-TOPSIS evaluation identified W2N2 (0.988) as the optimal regime, providing scientific support for efficient Zinnia cultivation and ecological-landscape coordination in arid oases.

## 1 Introduction

Zinnia (*Zinnia elegans Jacq.*) is an annual herbaceous ornamental flower of the genus Zinnia in the family Asteraceae (Qian et al., 2022). It is native to Mexico and is a typical heat-tolerant herbaceous flower in the south subtropical region during summer and autumn. It holds an important position in ornamental horticulture and is often referred to as one of the “Four Great Herbaceous Flowers” along with marigolds, petunias and salvi (Asif et al., 2024; Pan et al., 2024). The Zinnia flower is bright and beautiful, with exquisite shapes, early blooming and long flowering period. The dwarf Zinnia variety is suitable for container planting, while the tall variety is suitable for cut-flowers (Zhu et al., 2022). When planted alone, the dwarf Zinnia variety can form a purebred flower belt, while when combined with other flowers it can create a complex landscape with rich layers. In recent years, with the advancement of rural tourism, Zinnia, as an emerging ornamental plant, has become a dominant crop in the construction of “Beautiful Village”, playing a proactive role in supporting the development of modern agriculture and facilitating rural revitalization (Liu et al., 2025). The cultivated varieties of Zinnia had also gradually expanded from traditional monochromatic types to those with complex colors and gradient colors (Ali et al., 2021).

From the perspective of plant physiology, water serves as the material foundation in sustaining all the plant physiological and metabolic activities, not only providing structural support for cells but also acting as a medium for nutrient transport as well as a solvent for metabolic reactions throughout these processes (Eltarabily et al., 2019; Zarif et al., 2025). In addition, nitrogen is a critical nutrient element for the synthesis of core biological macromolecules such as proteins and nucleic acids, directly governing the construction and functional realization of biological organisms (Plett et al., 2020). During the entire plant growth and development cycling the dynamic balance between water and N persists, and their synergistic interaction is indispensable for the normal progression of life activities from germination to flowering (Chen et al., 2024; Wang et al., 2023b). In horticultural flower cultivation practices, the appropriate water conditions is a prerequisite for ensuring the active absorption of nutrients by roots, further providing an environmental basis for the efficient operation of core physiological processes such as the photosynthesis and respiration (Li et al., 2024; Panda et al., 2020). Studies on Zinnia had demonstrated that the balanced water and nitrogen supply not only ensured the stable operation of its photosynthetic system and the efficient translocation of plant nutrients, but also exerted a key regulatory effect on the ornamental trait formation including leaf expansion, the timing of flower bud differentiation, and the intensity of flower color (Elhindi et al., 2016). Experiments conducted by Fadhil et al (Fadhil et al., 2024). further confirmed that when water supply was deficient, Zinnia would exhibit growth abnormalities such as plant dwarfism, leaf chlorosis and yellowing, a sharp reduction in flower number and flower shape malformation, providing an inverse evidence for the core impact of water regulation on ornamental flower growth status.

The arid region of Northwest China is constrained by unique geographical and climatic conditions including its inland location and scarce precipitation, resulting in a particularly prominent water shortage issue. Additionally, long-term wind erosion had led to soil organic matter deficiency and generally low fertility levels (Hazbavi et al., 2020; Kang et al., 2025). The Zinnia plant has a certain drought tolerance compared to common ornamental flowers, with its photosynthetic system activity significantly suppressed under the extreme arid conditions in this region, accompanied by a sharp decline in the photosynthetic rate (Toscano and Romano, 2021). Concurrently, the accumulation processes of osmotic adjustment substances including the soluble proteins and soluble sugars and energy-storage nutrients were disrupted, ultimately leading to a deterioration of ornamental traits such as reducing the flower size and faded coloration as well as a simultaneous weakening of ecological functions involving sand fixation and water conservation (Chen et al., 2018; Kukuh et al., 2025). This region-specific challenge underscores the critical importance of precise water and nitrogen management in Zinnia cultivation within the arid northwest China. The irrigation and fertilization regimes tailored to the regional characteristics not only directly enhance soil water retention capacity and nutrient supply levels but also will promote targeted plant growth and development by regulating plant physiological metabolic processes such as proline synthesis and stomatal conductance (Liao et al., 2025; Wahocho et al., 2016). Notably, such targeted regulatory measures in an arid environment will stimulate plant roots to elongate into the deeper soil layers, improve the plant’s ability to acquire limited water and nutrient resources, optimize the carbon-nitrogen metabolism, improve the photosynthetic efficiency, ultimately achieving robust plant growth and significantly enhancing the flower quality (Abdelghany et al., 2025; Chen et al., 2025). Thus, the research and development of water and nitrogen management technologies aiming at soil fertility improvement has become a key priority in the arid northwest China. The scientific water and nitrogen regulation models can not only maximize the water and nutrient use efficiency of Zinnia but also effectively mitigate the dual inhibitory effects of drought stress and soil infertility, providing highly targeted technical support for ecological restoration and urban greening initiatives in the arid northwest China.

Specifically, our experiment was carried out in the cold and arid irrigation area of Hexi oasis where water resources were extremely scarce. Therefore, the objective of present study was to determine: (1) This study investigates the effects of water-nitrogen combination across distinct growth stages on Zinnia’s growth performance, leaf photosynthetic traits, plant physiological and biochemical parameters, soil nutrient dynamics, and ornamental quality (including floral morphology and color expression). (2) the optimal water and nitrogen management strategies through comprehensive multi-objective assessment. Consequently, the optimal ornamental effect of Zinnia under the lowest water and nitrogen input is to provide some scientific basis and technical support for urban greening projects and ecological construction in the arid areas of northwest China.

## 2 Materials and methods

### 2.1 Experimental site description

**Figure 1:**
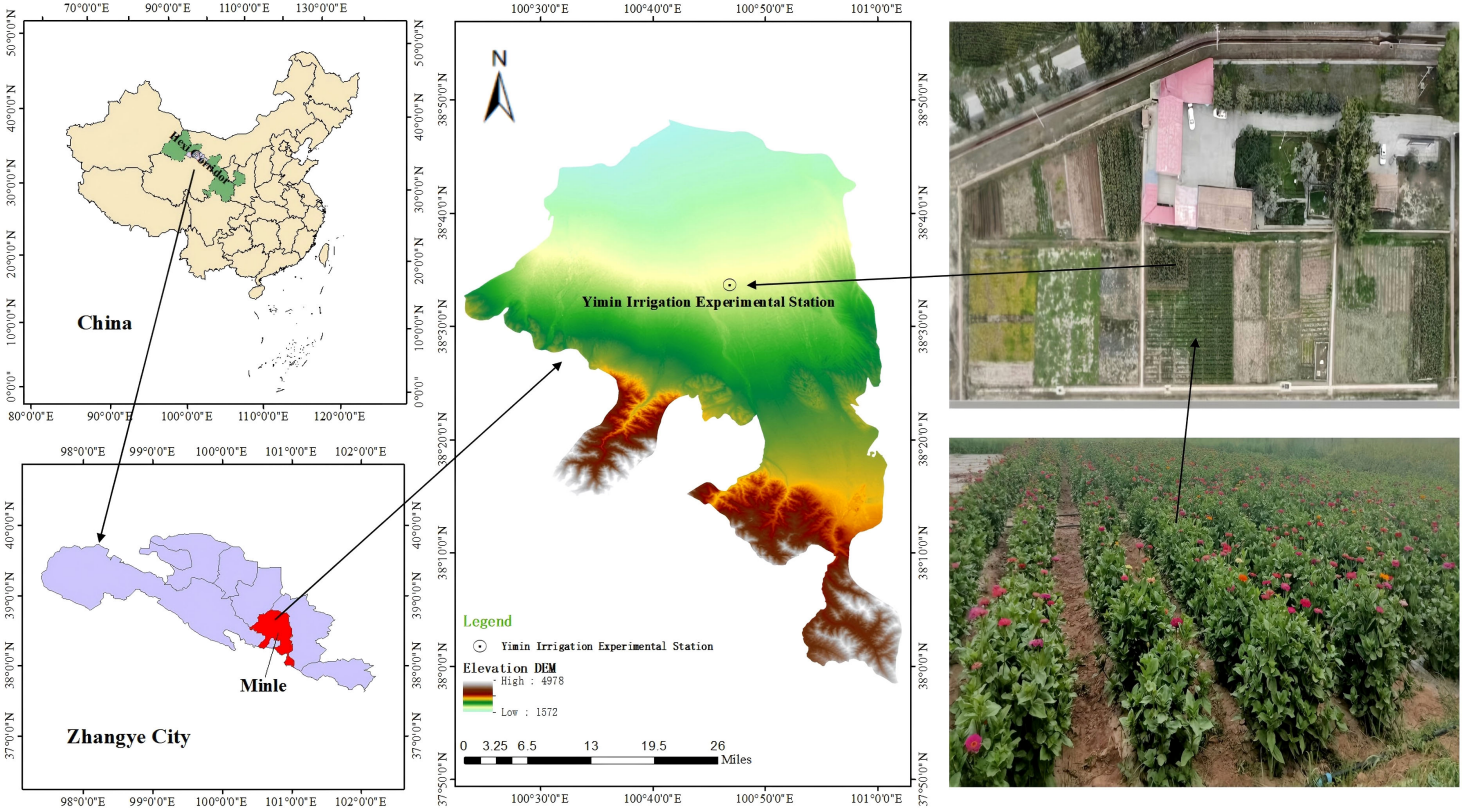
Study site location and Zinnia field experiment.

The field experiment was conducted in two consecutive seasons from May to September in 2024 and 2025 at the Yimin Irrigation Experiment Station in Zhangye City, Gansu Province, China (100°47′6″ E, 38°35′41″ N, 1977 m a.s.l.) on the same experimental plot (**Figure 1**) (Chen et al., 2025). The study area has a continental desert steppe climate with drastic temperature changes and scarce water resources, where the annual average temperature is 6°C, with the extreme highest temperature reaching 37.8°C and the extreme lowest temperature being -33.3°C, the total annual rainfall ranging from 183 to 285 mm, the frost-free period of 109 to 174 days, and the annual sunshine duration of approximately 3000 hours (Li et al., 2025).

**Figure 2:**
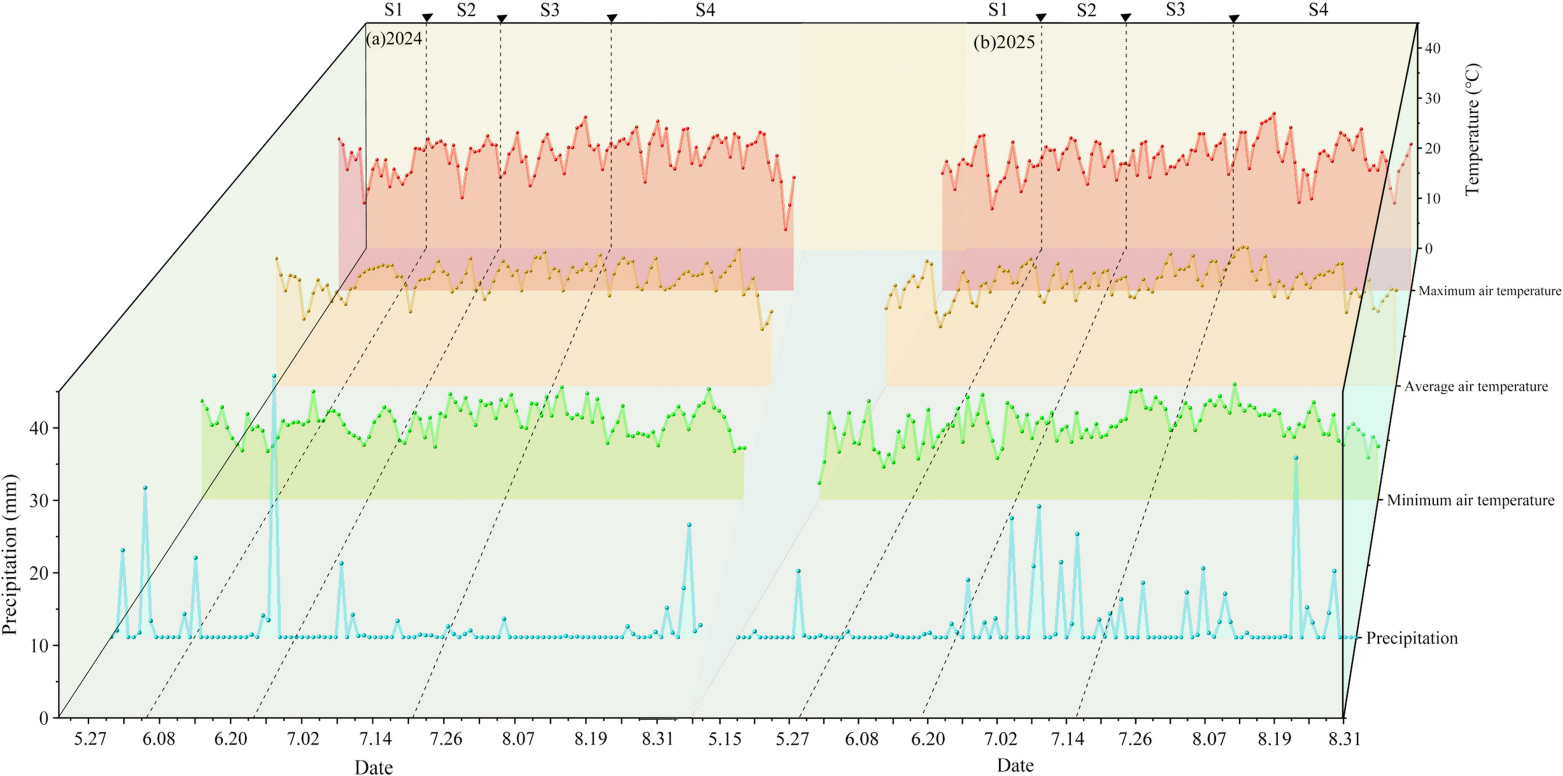
The precipitation and temperature during the Zinnia growth season (May to September) at the experimental site in 2024 **(a)** and 2025 **(b)**. S1, seedling; S2, budding; S3, early flowering; S4, full flowering.

A meteorological station of the Minle County Meteorological Bureau of Zhangye City was set up around the experimental field. During the experiment, meteorological data were automatically recorded by a meteorological observation station (DZZ6 type, Zhonghuan Tianyi) established beside the experimental field, with a recording interval of 1 hour. **Figure 2** show the daily variation patterns of various meteorological factors during the growth seasons of Zinnia in 2024 and 2025. As shown in the figures, the rainfall during the two growth seasons of Zinnia was relatively low, only 158 mm and 201.3 mm respectively, and mainly occurred in June, July, and August. Among them, there were three rainfall events exceeding 15 mm in each of the two growth seasons. The average daily temperature during the 2024 and 2025 growth seasons generally showed an increasing trend followed by a decreasing one. The temperature was relatively high in June, July, and August, and relatively low in May and August. The average temperatures of the two growth seasons were 18.96 °C and 18.44 °C respectively (**Figure 2**).

### 2.2 Experimental design

**Figure 3:**
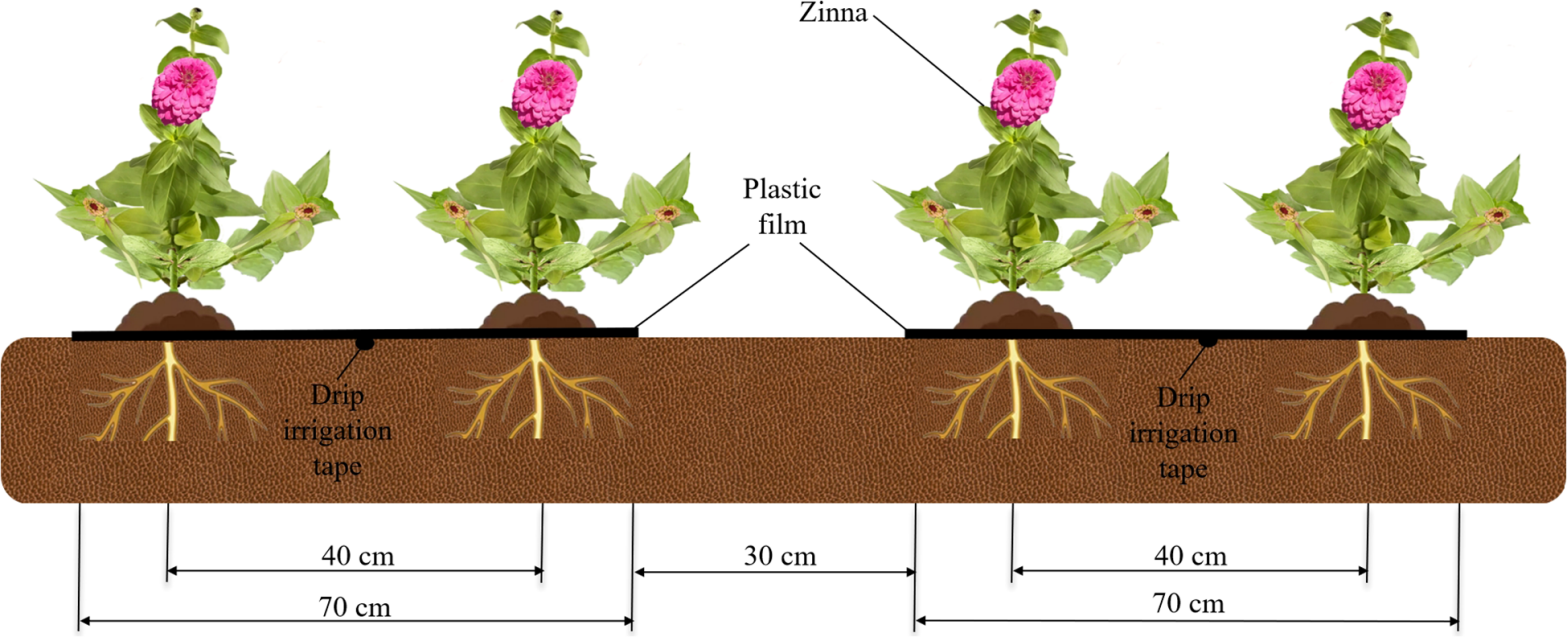
Cross-section of the Zinnia planting.

After variety adaptability screening, the “Pink Dream” series was selected as the Zinnia variety suitable for planting in the study area, which had distinct cultivation prerequisites of approximately 10 cm flower diameter, optimal growth temperature ranging from 20 to 35°C, good adaptation to the summer climate, early blooming, and excellent planting in summer. The selected experimental tube material for drip irrigation system was PE hose which was divided into three levels of pipelines including the main pipes, branch pipes, and capillary pipes with an inline labyrinth structure. The drip tapes were laid along the east-west direction with a spacing of 90 cm, using disposable specifications in a pipe diameter of 16 mm, an emitter spacing of 30 cm, a designed emitter flow rate of 3.0 L·h^-1^, and an operating pressure of 10 m water head. The experimental setup used a planting configuration of one drip irrigation tape under film-mulching displayed in the middle of two rows of Zinnia crops, maintaining a 20 cm distance from each row, with a single drip irrigation tape. The row spacing within the film is 40 cm and the plant spacing is 30 cm. (**Figure 3**) (Chauhdary et al., 2024; Donadkar and Fatmi, 2022).

**Figure 4:**
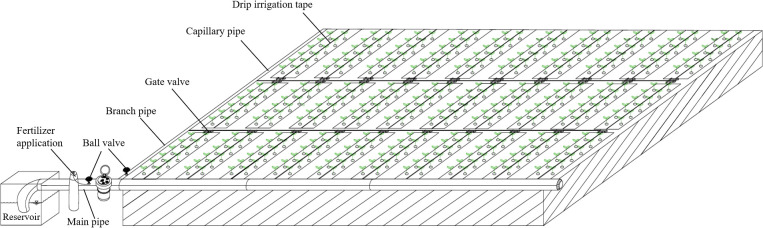
Schematic diagram of field layout.

Nine water-nitrogen combination treatments and one CK were established in this experiment, with three replicates, totaling 30 experimental plots measuring 9.0 m × 1.1 m in size. The total area of the experimental site was 297 m² (0.0445 hm²), and the planting density was uniformly set at 60000 plants·ha^-^¹. To prevent lateral water infiltration between plots, plastic film was installed at a depth of 0.6 m along plot boundaries. A drip irrigation tape mulched with film was adopted in each plot (**Figure 4**). The determination of each irrigation event is based on the soil moisture content of the planned wetting layer. Specifically, when the relative soil moisture content of the planned wetting layer is detected to be lower than or close to the preset design lower limit, the irrigation operation is immediately initiated. The required irrigation volume is precisely calculated strictly according to the preset design upper limit to ensure that the relative soil moisture content of the planned wetting layer exactly reaches the design upper limit after irrigation, meeting the water requirements of zinnia at different growth stages while avoiding excessive irrigation that could lead to waterlogging, root rot or water waste in the soil. The entire growth period of Zinnia was divided into four critical growth stages: seedling, bud formation, initial flowering, and full blooming. As shown in **Table 1**, soil moisture regulation was set at three gradients as follows: moderate deficit (W1, soil moisture maintained at 55% to 65% in field capacity, FC), mild deficit (W2, 65% to 75% in FC), and no water deficit (W3, 75% to 85% in FC). Four nitrogen application levels were implemented as following: low nitrogen application (N1, 90 kg·ha^-1^), medium nitrogen application (N2, 150 kg·ha^-1^), high nitrogen application (N3, 210 kg·ha^-1^), and local conventional nitrogen application (CK, 270 kg·ha^-1^). 40% of total nitrogen fertilizer was applied as base fertilizer, while the remaining 60% was equally distributed and applied accompanied with drip irrigation at bud formation, initial flowering, and full blooming of Zinnia, respectively. Phosphorus, potassium, and organic fertilizers were also uniformly applied as base fertilizers in a single application.

**Table 1:** Experimental design.

Treatment	N (kg·ha^-1^)	Soil water deficit level (%)			
		Seedling	Budding	Early flowering	Full flowering
W1N1	90	75∼85^a^	75∼85	75∼85	75∼85
W1N2		75∼85	75∼85	75∼85	75∼85
W1N3		75∼85	75∼85	75∼85	75∼85
W2N1	150	75∼85	75∼85	65∼75	75∼85
W2N2		75∼85	75∼85	65∼75	75∼85
W2N3		75∼85	75∼85	65∼75	75∼85
W3N1	210	75∼85	75∼85	55∼65	75∼85
W3N2		75∼85	75∼85	55∼65	75∼85
W3N3		75∼85	75∼85	55∼65	75∼85
CK	270	75∼85	75∼85	75∼85	75∼85

The lower and upper limit of soil water moisture contents in field capacity.

### 2.3 Measurements and calculations

#### 2.3.1 Soil nutrients

At the end of each Zinnia growth stage, three sampling locations were established per plot. At each sampling locations, two adjacent plants under similar growth conditions were randomly selected, and soil samples were collected within 0∼20 cm, 20∼40 cm, and 40∼60 cm depths between the two adjacent plants. Three soil samples from the same depth layer were mixed to form a composite sample after removing the impurities, then sieved through a 2 mm sieve for analysis. Total nitrogen (TN), and organic matter (SOM) content in each plot were determined using the semi-micro Kjeldahl method, and the potassium dichromate method, respectively, calculated according to the following formulas (Equation 1) (Hood-Nowotny et al., 2010; Pan et al., 2013; Qiu et al., 2026):

(1) $$\text{TN}=\frac{\text{(V}-{\text{V}}_{0})\times \text{c}\times 0.014\;\times 10^{3}}{\text{m}}$$

Where *V* the volume of the acid standard solution used during titrating (mL), *V_0_* represents the volume of the acid standard solution used during blank titrating (mL), *c* represents the concentration of the standard solution (mol·L^-1^), *0.014* represents the molar mass of nitrogen (kg·mol^-1^), *10³* represents the unit conversion factor.

#### 2.3.2 Soil enzyme activity

After soil sampling in the previous step, soil urease activity, catalase activity, and sucrase activity were determined using the phenol-sodium hypochlorite colorimetric method, potassium permanganate titration method, and 3,5-dinitrosalicylic acid colorimetric method, respectively, calculated according to the following formulas (Equations 2–4) (Burns et al., 2013; Nannipieri et al., 2018):

(2) $$\text{Ure}=\frac{{\text{(A}}_{\text{sample}}+{\text{A}}_{\text{soil free}}+{\text{A}}_{\text{substrate free}})\times \text{V}\times \text{n}}{\text{m}}$$

(3) $$\text{X}=\frac{{\text{(V}}_{0}-\text{V)}\times \text{T}}{\text{m}\times \text{f}}$$

(4) $$Suc=\frac{a\times V\times n}{m}$$

where *Ure* represents soil urease activity (mg·g^-1^·24h^-1^), *X* represents catalase activity, *Suc* represents soil sucrase activity, A sample represents the absorbance of the sample reaction solution (mg), A soil free represents the absorbance of the soil-free control solution (mg), A substrate free represents the absorbance of the substrate-free control solution, *V* represents the volume of the chromogenic liquid or the volume of the potassium permanganate standard solution consumed in titration for the sample. *V_0_*represents the volume of the acid standard solution used for titrating the blank (ml), *T* represents the correction value of the potassium permanganate titration, *f* represents the conversion factor of the titrant (mg·ml^-1^), *a* represents the glucose concentration in the reaction solution (mg·ml^-1^), *n* represents the dilution multiple of the reaction solution, and m represents the mass of the soil sample (g).

#### 2.3.3 Physiological and biochemical indices

During the Zinnia budding and full flowering, three representative plants were selected from each plot for flower and leaf sampling. The samples were immediately sealed in plastic self-sealing bags and placed in a portable frozen sampling box, promptly stored in a -20°C refrigerator. Subsequently, the determination of physiological and biochemical indicators was completed in the indoor laboratory. The determinations were carried out using the spectrophotometry, Coomassie Brilliant Blue G-250 staining method and anthrone colorimetry, respectively. Each index was calculated according to the following formulas (Equations 5, 6) (Gupta et al., 2015; Özgür and Çimen, 2018; Wang et al., 2024):

(5) $$ANT/Lutein/SP=\frac{m\times V_{T}\times N}{m_{0}\times V_{s}\times 1000}$$

(6) $$SS=\frac{m\times V_{T}\times N}{m_{0}\times V_{s}\times 10^{6}}\times 100$$

where *m* represents the concentration of protein, sugar, anthocyanin, and Lutein in the test solution as determined by the standard curve (μg·g^-1^); *V_T_* represents the total volume of the sample extraction (ml); *m_0_* represents the mass of the sample (g); *V_s_* represents the volume of the sample supernatant used for testing (ml); and *N* is the dilution factor.

#### 2.3.4 Zinnia ornamental value

During the full flowering stage of Zinnia, 3 Zinnia plants with similar growth vigor were randomly selected in each plot, and the number of flowers was picked per batch, and the number of lateral branches were recorded, respectively. A CJW888 digital display vernier caliper was used to measure the diameter of the largest flower in each plant, and the number of petals was recorded.

#### 2.3.5 Zinnia growth indexes

At the end of each Zinnia growth stage, six plants exhibiting uniform and vigorous growth were selected per plot to determine growth dynamic indices systematically. The plant height was measured using a steel tape, the stem diameter was determined using a CJW888 digital display vernier caliper. Leaf area was determined by the hole-cutting method: representative functional leaves were selected at the critical growth stage, and several uniform circular leaf pieces were cut with a hole puncher of known area, avoiding the leaf veins. The leaf pieces were dried to a constant weight and weighed (Mason et al., 2012). The specific leaf weight (SLW) was calculated based on the area of the leaf pieces and their dry weight. Then, the total leaf area per plant was estimated based on the total dry weight of the leaves per plant and SLW (Mason et al., 2012). Finally, the leaf area index (LAI) was calculated by combining the total leaf area per plant with the number of plants per unit area (Mason et al., 2012). Following the onset of the full-bloom stage in Zinnia, destructive sampling was performed at 20-day intervals. Specifically, three representative 1 m × 1 m subplots were randomly selected from each experimental plot, and the aboveground dry matter accumulation (DMA) was determined using the oven-drying method (Alefu et al., 2023).

#### 2.3.6 Cut flower yield

When the plants reached the late growth stage, all harvestable flowers had achieved physiological maturity, characterized by plump floral morphology, fully expanded petals, and healthy, upright flower stems. No unopened buds or flowers failing to meet the harvest criteria were observed (R et al., 2025). Under these strict harvest standards, a one-time full-plant harvest is carried out to ensure that all cut flowers obtained are 100% in line with commercial harvest specifications, and to prevent any omissions or excessive picking (Deng et al., 2024). After the harvest is completed, the cut flowers are immediately packaged separately by plot number to strictly avoid cross-mixing (Ngubeni and Wahome, 2022). Then, the total fresh weight of the cut flowers in each plot is measured using an electronic balance with a precision of 0.01 g. All weighing data are independently entered and cross-checked by two people (for a total of two rounds) (Chen et al., 2024). Only after confirming that there are no omissions in the records, unit errors, or calculation deviations are the data considered as the final experimental data.

#### 2.3.7 Photosynthetic parameters

At the end of each growth stage of Zinnia, measurements were conducted on clear, cloudless days using a LI-6400 Portable Photosynthesis System (LI-COR Biosciences, Lincoln, NE, USA). Between 8:00 and 16:00, three plants were randomly selected from each experimental plot. Photosynthetic parameters (net photosynthetic rate, Pn; transpiration rate, Tr) were measured at the mid-section of fully expanded functional leaves, sampled from the top to bottom of each plant, with three replicate measurements per plant. During measurements, leaves were unobstructed and exposed to direct sunlight. For the determination of LAD and NAR, three representative 1 m × 1 m subplots were randomly selected from each experimental plot. Destructive or non-destructive sampling was performed to measure leaf area index LAI and DAM. The photosynthetic potential and net assimilation rate of Zinnia were then calculated based on the aforementioned data, using the formulas provided below (Equations 7–9) (Deng et al., 2024; Narayan et al., 2023):

(7) $$LAI=\frac{LA\times D}{A}$$

(8) $$\text{LAD}=\frac{{\text{LA}}_{1}+{\text{LA}}_{2}}{2}\times ({\text{t}}_{2}-{\text{t}}_{1})$$

(9) $$\text{NAR}=\frac{{\text{(lnLA}}_{2}-{\text{lnLA}}_{1}{\text{)(W}}_{2}-{\text{W}}_{1})}{{\text{(LA}}_{2}-{\text{LA}}_{1}{\text{)(t}}_{2}-{\text{t}}_{1})}$$

Where *LAI* represents the leaf area index of Zinnia (m²·plant^-1^), *LAD* represents the photosynthetic potential of ornamental sunflower (10^4^·m^2^·d·ha^-1^), *NAR* represents the net assimilation rate of ornamental sunflower (g·m^-2^·d^-1^), *LA_1_* and *LA_2_* represent the leaf area measured at times *t_1_* and *t_2_* (10^4^·m²·ha^-1^), *t_1_* and *t_2_* are the dates corresponding to the two consecutive measurements (d), *W_2_* and *W_1_* are the plant dry weights measured at *t_2_* and *t_1_* (g), and *A* is the land area (m²).

#### 2.3.8 Comprehensive evaluation

In the multi-index comprehensive evaluation process, determining indicator weights and ranking evaluation objects are critical steps that underpin the scientific rigor and reliability of evaluation outcomes. To mitigate biases introduced by subjective weighting and enhance the objectivity and accuracy of comprehensive evaluation results, this study employs the entropy-weighted TOPSIS method for integrated assessment. The entropy-weighted TOPSIS method is a multi-attribute decision-making analysis method that combines the entropy weight method and the TOPSIS method. First, this method uses the entropy weight method to determine the weights of each indicator based on the magnitude of their information entropy, avoiding the arbitrariness of subjective weighting and improving the objectivity of the evaluation. Subsequently, through the TOPSIS method (Technique for Order Preference by Similarity to Ideal Solution), each evaluation object is ranked to identify the optimal and worst solutions, thereby determining the relative superiority or inferiority of each evaluation object. It includes the following steps (Mahammad and Islam, 2024; Narayan et al., 2023):

(1) Construct a standard matrix (Equation 10)

(10) $$X_{ij}=\frac{{\text{x}}_{ij}-{\text{x}}_{\min}}{{\text{x}}_{\max}-{\text{x}}_{\min}}$$

In the formula, *x_ij_* (where *i* = 1, 2,…, m, representing the i-th treatment; *j* = 1, 2,…, n, representing the j-th indicator).

(2) Calculate the feature proportion (P_ij_) of the i-th evaluation processing under the j-th indicator (Equation 11)

(11) $${\text{P}}_{\text{i}\text{j}}\;=\frac{{\text{X}}_{\text{i}\text{j}}}{\sum_{\text{I}=1}^{\text{n}}{\text{X}}_{\text{i}\text{j}}}$$

(3) Calculate the information entropy value (E_ij_) of the j-th indicator (Equation 12)

(12) $$\;\text{E}\text{i}\text{j}=\;-\text{K}\;\times \sum_{\text{i}=1}^{\text{n}}\left({\text{P}}_{\text{i}\text{j}}\;\times \text{ln}({\text{P}}_{\text{i}\text{j}})\right)$$

where K = 1ln(m), ensuring that the value range of E_j_ is between 0 and 1

(4) Calculate the difference coefficient ( ${\text{D}}_{\text{j}}$) of the j-th indicator (Equation 13)

(13) $$D_{j}\;=1-E_{j}\;$$

(5) Calculate the entropy weight (W_j_) of the j-th indicator (Equation 14)

(14) $$Wj\;=\frac{d_{j}}{\sum_{j=1}^{m}d_{j}}$$

(6) Standardization of the original matrix (Equation 15)

(15) $$Z_{ij}=\frac{x_{ij}}{\sqrt{\sum_{i=1}^{m}x_{ij}^{2}}}$$

In the formula, *z_ij_* (where i = 1, 2,…, m, represents the i-th treatment after standardization; and j = 1, 2,…, n, represents the j-th indicator after standardization)

(7) Construct a weighted matrix (Equation 16)

(16) $$R=W_{j}\times Z_{ij}$$

(8) Construct the positive ideal solution (R^+^) and the negative ideal solution (R^-^) (Equations 17, 18)

(17) $${\text{R}}^{+}=\;({\text{R}}_{\text{max}\;1},{\text{R}}_{\text{max}\;2},\ldots ,{\text{R}}_{\text{max}\;3})$$

(18) $${\text{R}}^{-}\;=\;({\text{R}}_{\text{min}\;1},{\text{R}}_{\text{min}\;2},\ldots ,{\text{R}}_{\text{min}\;3})$$

(9) Calculate the Euclidean space distance from each evaluated object to the positive ideal solution ( $D_{I}^{+}$) and the negative ideal solution ( $D_{I}^{-}$) (Equations 19, 20)

(19) $$D_{i}^{+}=\sqrt{\sum_{j=1}^{n}{\left(R_{ij}-R_{ij}^{+}\right)}^{2}}\;$$

(20) $$D_{i}^{+}=\sqrt{\sum_{j=1}^{n}{\left(R_{ij}-R_{ij}^{-}\right)}^{2}}\;$$

(10) Calculate the closeness degree (C_i_) between each evaluation index and the optimal scheme (Equation 21)

(21) $$C_{i}=\frac{D_{i}^{-}}{D_{i}^{+}+D_{i}^{-}}\;$$

### 2.4 Statistical analysis

The indicators in this study represent the average data of three repeated experiments. Data organization was completed using Excel 2019 (Microsoft Corporation, United States). A two-way analysis of variance (ANOVA) was performed with IBM SPSS Statistics 27 software (IBM Corporation, United States) on the indicators over two years. Irrigation level and N fertilizer application rate were used as fixed factors to evaluate their main effects and interaction effects (W×N). Mean comparisons among treatments were conducted using Duncan’s multiple range test at a significance level of *P* < 0.05. Graphs were created using software such as Origin 2025b (OriginLab Corporation, Northampton, Massachusetts, United States).

## 3 Results

### 3.1 Soil environment

#### 3.1.1 Soil total nitrogen content

**Figure 5:**
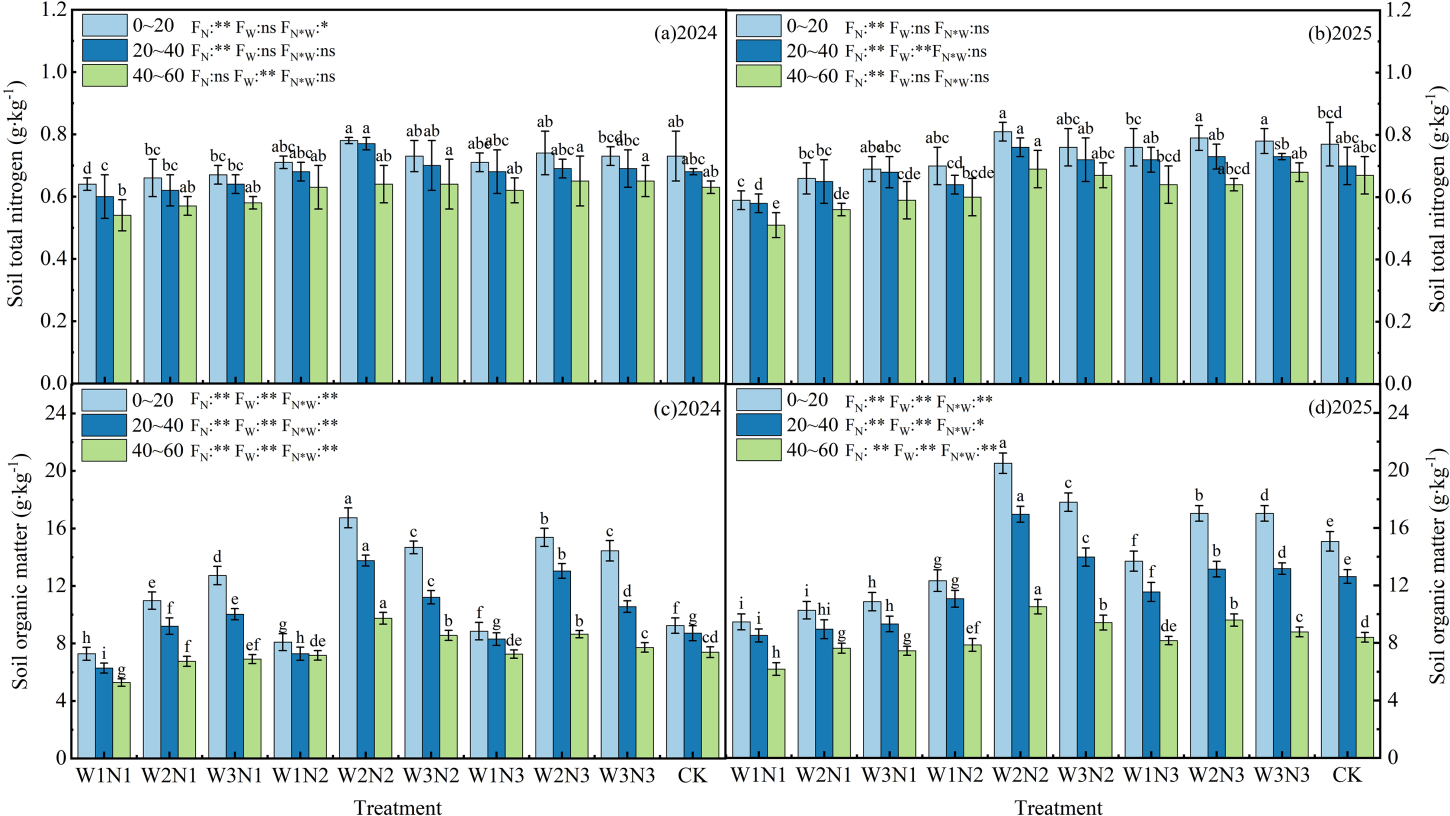
The effect of different irrigation methods and N fertilizer application schemes on soil TN and SOM content (0∼60 cm) of Zinnia in 2024 **(a, c)** and 2025 **(b, d)**. Different lowercase letters indicate significant differences between treatments at the *P* < 0.05 level. The error bar indicates the standard deviation. ** indicates significant differences at the *P* < 0.01 level, and ns indicates no significant difference (P > 0.05).

**Figures 5a,b** shows the effects of different irrigation and nitrogen application treatments on the TN content of the soil in the 0∼60 cm plough layer of Zinnia during two growing seasons. The analysis of variance indicated that the irrigation levels and nitrogen application rates in 2024 and 2025 had no significant (*P* > 0.05) effect on the TN content of the soil, and the interaction effect between the two was also not significant (*P* > 0.05). As shown in the figure, in 2024, under different water and nitrogen treatments, the TN content of the soil in the 0∼20 cm, 20∼40 cm, and 40∼60 cm layers was respectively between 0.64 to 0.78 g·kg^-1^, 0.60 to 0.77 g·kg^-1^, and 0.54 to 0.65 g·kg^-1^; in 2025, the corresponding soil TN content in each layer was respectively between 0.59 to 0.81 g·kg^-1^, 0.58 to 0.76 g·kg^-1^, and 0.51 to 0.69 g·kg^-1^. Compared with the N1 treatment, the TN content of the soil in the two years under the N2, N3, and N4 treatments showed a slight upward trend. In 2024, the increase in the 0∼20 cm, 20∼40 cm, and 40∼60 cm layers was respectively between 14.06% to 21.88%, 13.23% to 28.33%, and 16.67% to 20.37%; in 2025, the increase in each layer was respectively between 10.17% to 37.29%, 20.69% to 31.03%, and 31.37% to 35.29%. With the increase in irrigation levels, the TN accumulation in the soil at the N1 level showed an upward trend, while at the N2 and N3 levels, it showed an initial increase followed by a decrease. Moreover, there were significant (*P* < 0.05) differences between W1 and W2, W3 treatments, but no significant (*P* > 0.05) difference between W2 and W3 treatments. In summary, under different nitrogen application levels, an increase in soil depth had a significant negative impact on the TN content in the 0∼60 cm plough layer of Zinnia. The TN accumulation effect in the surface soil was much higher than that on the deep soil. Additionally, the application of additional nitrogen fertilizer had a certain positive enrichment effect on the TN in each soil layer, but due to the influence of experimental factors, this effect did not reach a significant level (*P* > 0.05).

#### 3.1.2 Soil organic matter content

**Figures 5c,d** show the effects of different irrigation and nitrogen application treatments on SOM in the plough layer (0-60 cm) of Zinnia during two growing seasons. The analysis of variance indicated that the irrigation level, nitrogen application rate, and their interaction significantly affected the soil TN content in 2024 and 2025 (*P* > 0.05). The SOM content exhibited a consistent vertical distribution pattern with total nitrogen. In 2024, the organic matter content in the 0∼20 cm, 20∼40 cm, and 40∼60 cm soil layers ranged from 7.28 to 16.74 g·kg^-1^, 6.28 to 13.76 g·kg^-1^, and 5.28 to 9.75 g·kg^-1^, respectively; in 2025, the corresponding values were 9.46 to 20.50 g·kg^-1^, 8.52 to 16.95 g·kg^-1^, and 6.18 to 10.52 g·kg^-1^, respectively. The SOM content in 2025 was generally higher than that in 2024 at the same soil layer, and the organic matter content in the 0∼20 cm surface soil was the highest among all treatments, while that in the 40∼60 cm layer was the lowest. The differences among soil layers were significant, but the fluctuations in organic matter content within the same soil layer under different water and nitrogen treatments were relatively small, with no obvious irrigation or nitrogen application gradient change pattern. As shown in **Figure 5**, in each soil layer, compared with N1, the SOM content under N2 and N3 treatments showed a significant (*P<* 0.05) upward trend in both years. In 2024, the increase in SOM content in each soil layer ranged from 11.95% to 129.94%, 14.65% to 119.11%, and 8.14% to 84.66%, respectively; in 2025, the increase ranged from 11.29% to 116.67%, 10.09% to 98.94%, and 7.28% to 70.23%, respectively. This indicates that the surface soil was most affected by nitrogen application, and the SOM content in the surface soil showed a clear enrichment trend with increasing nitrogen application. Although the SOM content in the deep plough layer was also positively affected by increasing nitrogen application, the increase was less than that in the surface soil. With the increase in irrigation level, the SOM content increased at the N1 level, but showed an initial increase followed by a decrease at the N2 and N3 levels. In 2024, the SOM content in the 0∼20 cm soil layer reached its peak under the N2 treatment, and the SOM in the 20∼40 cm and 40∼60 cm soil layers was also higher under the N2 treatment than under the N1 and N3 treatments. The trend of TN accumulation in each soil layer in 2025 was basically the same as that in 2024. From the perspective of different irrigation treatments, the SOM content in each soil layer generally increased with the increase in irrigation volume, but the increase rate slowed down when the irrigation volume reached a certain level. In the 40∼60 cm soil layer, compared with the low nitrogen application treatment, the soil organic matter content under the medium and high nitrogen application treatments also showed a significant upward trend in both years. The increase in 2024 was between, and in 2025 it was between. The increase effect of the high nitrogen application treatment was still relatively significant, indicating that the organic matter content in the deep plough layer was also positively affected by increasing nitrogen application, but the increase was less than that in the surface soil.

#### 3.1.3 Soil enzyme activity

**Figure 6:**
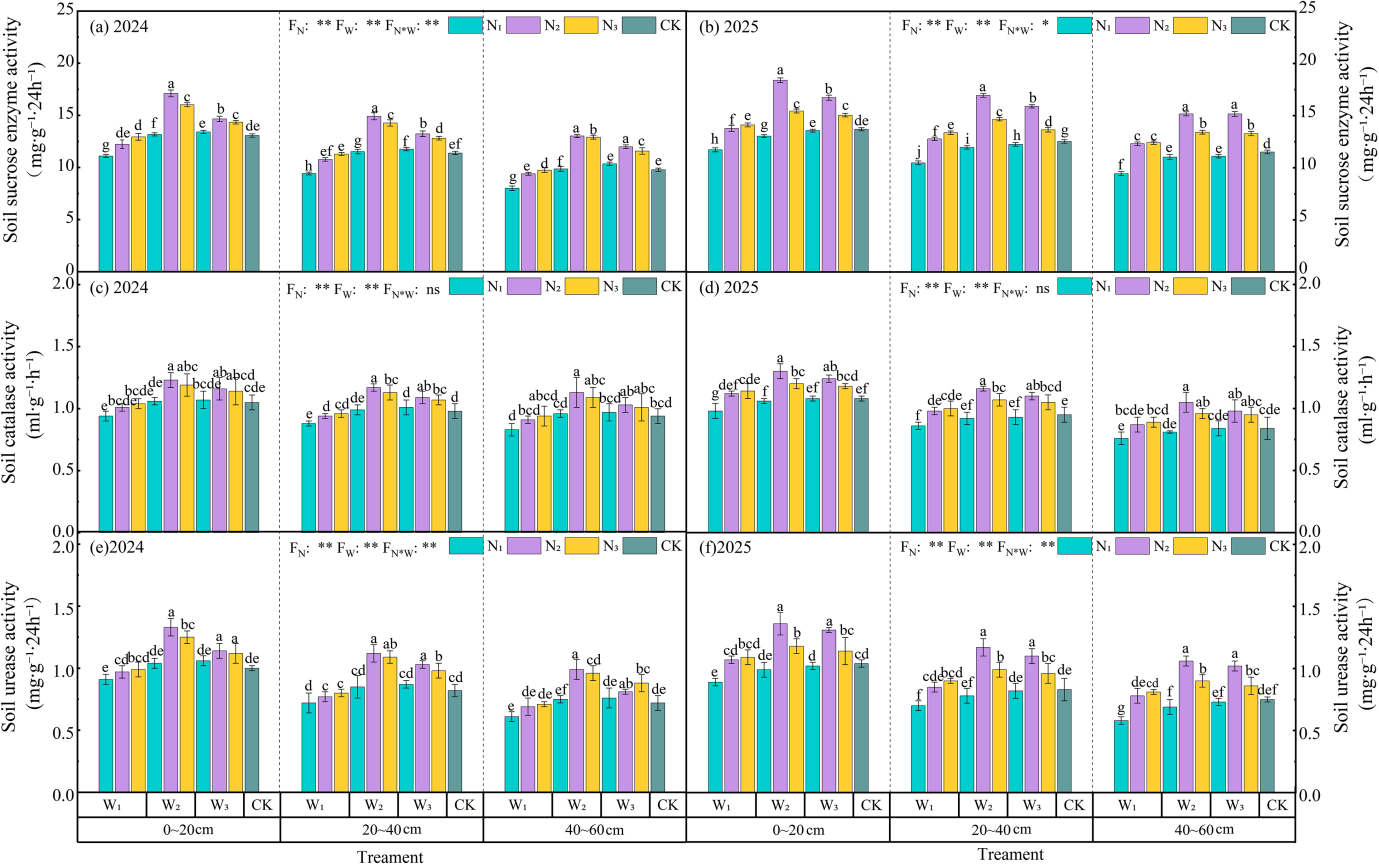
Soil enzyme activities under different irrigation methods and N fertilizer application treatments in 2024 **(a, c, e)** and 2025 **(b, d, f)**. Lowercase letters indicate significant differences among treatments (*P* < 0.05), and error bars represent standard deviations. *, **, and ns represent significant effects at the 0.05 level, 0.01 level, and no significant effect (*P* < 0.05), respectively.

As shown in **Figure 6**, different irrigation levels under the same N rate had distinct effects on the activities of soil urease, catalase, and sucrase. At the W1 irrigation level, compared with CK, the activities of soil urease, catalase, and sucrase under different N application rates decreased by 1.31% to 17.05%, 1.01% to 9.19%, and 1.51% to 19.64% over two years, respectively. At the W2 irrigation level, compared with CK, the application of N fertilizer at different rates resulted in an average increase of 2.96% to 27.02%, 1.33% to18.33%, and 0.95% to 23.98% in the three enzyme activities in 2024 and 2025, respectively. Among them, N2 and N3 treatments reached a significant (*P* < 0.05) level and a highly significant (*P* < 0.01) level compared with CK, respectively. Meanwhile, it should be noted that the impact on soil enzyme activities is reflected not only in the amount of N fertilizer but also in the irrigation volume. For example, after applying N1 level of urea to the soil samples, the activities of soil urease, catalase, and sucrase increased with the increase of irrigation volume. However, after applying N2 or N3 level of urea, the enzyme activities showed a trend of first increasing and then decreasing with the increase of irrigation volume. Among all treatments, the one with relatively high soil enzyme activity (W2N2) was significantly (*P* < 0.05) superior to CK. Additionally, the enzyme activity was the highest in the plow layer soil (0∼20 cm) and decreased with the increase of soil depth.

### 3.2 Physiological and biochemical indices

**Figure 7:**
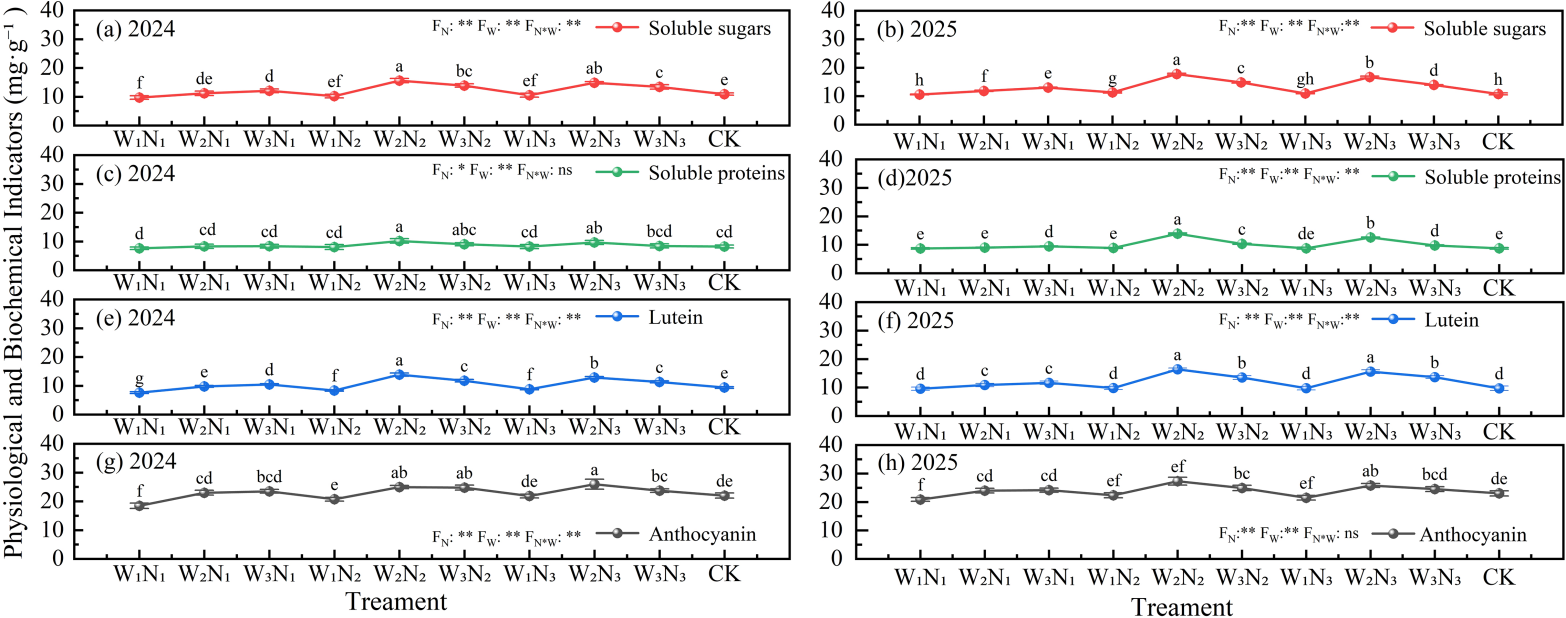
The effect of different irrigation methods and N fertilizer application schemes on physiological and biochemical indices of Zinnia in 2024 **(a, c, e, g)** and 2025 **(b, d, f, h)**. Different lowercase letters indicate significant differences between treatments at the *P* < 0.05 level. The error bar indicates the standard deviation. *, **, and ns represent significant effects at the 0.05 level, 0.01 level, and no significant effect (P< 0.05), respectively.

Among them, anthocyanin mainly imparts red, purple, and blue hues to the flowers, while lutein dominates the yellow and orange phenotypes (**Figures 7a–d**). The two-year experimental data from 2024 to 2025 showed that the anthocyanin content in the petals of pink dream Zinnia was significantly (*P* < 0.05) higher than that of lutein, confirming that anthocyanin is the key pigment determining the color of this cultivar. Water and N regulation had a significant regulatory effect on the contents of the two pigments, with the W2N2 and W2N3 treatments showing particularly prominent enhancement effects. In 2024, the anthocyanin content under these two treatments significantly increased by 11.78% to 17.86% compared with the CK, and the lutein content significantly increased by 14.43% to 23.54%. In 2025, the increase range of anthocyanin remained at 10.74% to 18.46%, while that of lutein further expanded to 37.08% to 67.31%, and the data from both years showed consistent significant (*P* < 0.05) differences.

Soluble sugars and soluble proteins, as important metabolic components of Zinnia, play a key role in plant growth and development as well as stress adaptation (**Figures 7e–i**). Monitoring results over two consecutive years showed that different water and N regulation treatments had a significant impact on the accumulation levels of the above two metabolic components. Among them, the W2N2 and W2N3 treatments exhibited a significant promoting effect. Compared with the CK, the comprehensive increase in soluble sugar and soluble protein content in the W2N2 and W2N3 treatments over these two years reached 14.43% to 59.21%, and the differences were all statistically significant (*P* < 0.05). This indicates that the synergistic regulation mode of W2 with N2 and N3 can effectively improve the carbon and N metabolic efficiency of Zinnia.

Studies have shown that appropriate water and N regulation can increase osmotic adjustment substances (soluble sugar, soluble protein), enhance the stress resistance of plants in stressful environments. With the increase of osmotic adjustment substances, the retention rate of anthocyanin and lutein in Zinnia also increases, thereby improving the ornamental value of Zinnia and ensuring better quality.

### 3.3 Growth indices

As shown in **Table 2**, in the two growth seasons of Zinnia, the irrigation level had a highly significant effect (P< 0.05) on plant height (PH), stem diameter (SD), dry matter accumulation (DAM), leaf area index (LAI), the number of lateral branches per plant (LBN), and the number of flowers per plant (NF). Among them, NF was mainly regulated by the genetic characteristics of the variety, and its response intensity to the irrigation level was slightly lower than that of the other four indicators. The amount of nitrogen fertilizer applied only had a highly significant (*P* < 0.05) effect on PH, DAM, and NF. The interaction between irrigation level and nitrogen fertilizer application (W×N) had a significant (*P* < 0.05) effect on PH, SD, DAM, LAI, and NF in both growth seasons. Further analysis indicated that this interaction significantly (*P* < 0.05) affected DAM, LAI, and NF in both years, but had no significant (*P* > 0.05) effect on PH and SD. Additionally, W×N had a significant (*P* < 0.05) effect on LBN in 2025 but did not reach a significant level in 2024 (P > 0.05).

**Table 2:** Effects of different irrigation methods and nitrogen application treatments on plant growth indices in 2024 and 2025.

Years	Treatment	PH (cm)	SD (mm)	LAI (m^2^·plant^-1^)	DMA (g·plant^-1^)	LBN (plant^-1^)	NF (plant^-1^)
2024	W1N1	112.73 ± 4.15e	18.01 ± 1.09ef	8.93 ± 0.53e	135.53 ± 3.91i	10.00 ± 2.00e	6.33 ± 2.52d
	W2N1	127.78 ± 3.03d	18.76 ± 0.66de	10.89 ± 0.83d	189.99 ± 4.22f	14.00 ± 3.00cde	11.67 ± 2.52bc
	W3N1	129.8 ± 2.05cd	18.81 ± 0.81d	12.17 ± 0.80c	209.05 ± 5.23e	14.67 ± 1.53cde	12.33 ± 1.15bc
	W1N2	116.98 ± 3.52bcd	18.21 ± 0.92f	9.38 ± 0.25de	139.71 ± 2.16i	12.00 ± 2.65de	8.00 ± 1.00cd
	W2N2	143.38 ± 5.66a	21.37 ± 1.17a	16.31 ± 0.75a	329.09 ± 3.27a	22.00 ± 3.00a	18.33 ± 5.03a
	W3N2	133.23 ± 2.04b	19.51 ± 0.82bc	13.67 ± 0.89b	248.11 ± 2.49c	17.67 ± 1.53abc	12.00 ± 2.00bc
	W1N3	123.26 ± 3.084d	18.52 ± 0.67e	9.65 ± 0.57cde	153.62 ± 3.75h	12.33 ± 3.0 6de	8.67 ± 1.53cd
	W2N3	137.95 ± 4.21bc	19.92 ± 0.66b	15.82 ± 1.20a	293.50 ± 2.66b	20. 67 ± 2.52ab	14.00 ± 3.3ab
	W3N3	132.68 ± 5.13bcd	19.27 ± 0.73bcd	13.48 ± 0.39b	233.94 ± 3.71d	16. 67 ± 1.53bcd	13.00 ± 1.00bc
	CK	125.62 ± 3.52bcd	18.65 ± 1.31e	10.59 ± 0.66cd	167.94 ± 3.22g	13.33 ± 3.06cde	10.67 ± 3.06cbcd
ANOVA	FN	**	ns	**	**	ns	**
(F value)	FW	**	**	**	**	**	**
	FN*W	ns	ns	**	**	ns	**
2025	W1N1	123.25 ± 1.94f	16.03 ± 1.56g	10.23 ± 0.38d	161.55 ± 2.56j	10.33 ± 1.53f	8.67 ± 1.53f
	W2N1	128.37 ± 3.91cd	18.16 ± 0.89de	12.76 ± 0.62c	236.32 ± 2.37f	18.33 ± 2.08cd	13.33 ± 3.06cd
	W3N1	130.21 ± 3.76c	18.83 ± 0.86d	13.00 ± 0.96c	257.32 ± 5.26e	12.67 ± 2.08bc	16.33 ± 1.53c
	W1N2	125.71 ± 2.83e	17.05 ± 0.93f	10.92 ± 0.30d	176.34 ± 3.17i	12.67 ± 2.08d	9.33 ± 2.08ef
	W2N2	140.04 ± 1.09a	20.03 ± 1.26a	17.30 ± 0.44a	409.79 ± 4.17a	25.00 ± 1.61a	28.33 ± 1.08a
	W3N2	133.57 ± 4.76bc	18.32 ± 1.83b	14.91 ± 0.74c	299.38 ± 3.23c	22.67 ± 3.51abc	20.33 ± 2.08b
	W1N3	126.61 ± 3.36e	17.33 ± 0.72e	11.15 ± 0.45d	194.91 ± 3.11h	15.33 ± 3.06de	10.67 ± 1.53def
	W2N3	137.62 ± 2.67ab	19.39 ± 1.25b	16.77 ± 1.33b	310.08 ± 4.86d	24.67 ± 1.53ab	22.33 ± 2.08ab
	W3N3	135.39 ± 1.12b	18.88 ± 1.31c	13.37 ± 0.67c	268.86 ± 3.27d	15.00 ± 1.00de	21.67 = 1.00b
	CK	127.94 ± 4.74d	17.61 ± 0.89e	12.71 ± 0.79c	225.40 ± 3.26g	13.00 ± 1.00ef	12.67 ± 1.53def
ANOVA	FN	**	ns	**	**	ns	**
(F value)	FW	**	**	**	**	**	**
	FN*W	ns	ns	**	**	**	**

PH, plant height (mm); SD, stem diameter (mm); LAI, leaf area index (leaf area per unit area) (m2·plant-1); DMA, dry matter accumulation of per unit area (g·plant-1); LBN, the number of lateral branches per plant (plant-1); NF, the number of flowers per plant (plant-1). Different letters indicate significant differences at the P< 0.05 level according to Duncan’s test. *, **, and ns represent significant effects at the 0.05 level, 0.01 level, and no significant effect (P< 0.05), respectively.

The results of two growing-season experiments showed that in 2024, the ranges of PH, SD, LAI, DAM, LBA, and NF were 112.73 to 143.38 cm, 18.01 to 21.37 mm, 6.50 to 16.31 m^2^·plant^-1^, 135.53 to 329.09 g·plant^-1,^ 10 to 22 plant^-1^ and 8 to 18.33 plant^-1^, respectively; in 2025, the corresponding ranges were 123.25 to 140.04 cm, 16.03 to 20.03 mm, 7.79 to 24.63 m^2^·plant^-1^, 161.55 to 409.79 g·plant^-1^, 10.33 to 25 plant^-1^, and 8.67 to 22.33 plant^-1^, respectively. Under the condition of constant nitrogen fertilizer application, the five growth indicators of Zinnia (PH, SD, LAI, DAM, LBA) in both 2024 and 2025 showed a significant (*P* < 0.05) upward trend with the increase of irrigation volume; although the NF was restricted by genetic characteristics, it still showed a continuous and stable growth trend with the improvement of irrigation conditions. Compared with the W1 level, the W2 level had the most significant effect on the improvement of all growth indicators, with an increase of 7.83% to 30.45% in 2024 and 8.25% to 31.72% in 2025. Under the condition of constant irrigation level, all growth indicators of the W1 treatment group continued to increase with the increase of nitrogen fertilizer application in both years; while all indicators of the W2 treatment group reached their peak at the N2 nitrogen application level in both years, and N2 was 3.10% to 30.93% and 1.76% to 32.16% higher than N3 treatment, respectively. Notably, NF was best in the N2 treatment at all irrigation levels. Combined with the analysis of meteorological data during the same period, it was speculated that there was a period of excessive precipitation during the flowering period in both years; under the condition of medium irrigation (W2) combined with high nitrogen (N3), excessive soil moisture content was likely to cause root hypoxia, thereby inhibiting vegetative growth and indirectly restricting the normal development of flower organs.

### 3.4 Ornamental value

#### 3.4.1 Main flower diameter and petal number

**Figure 8:**
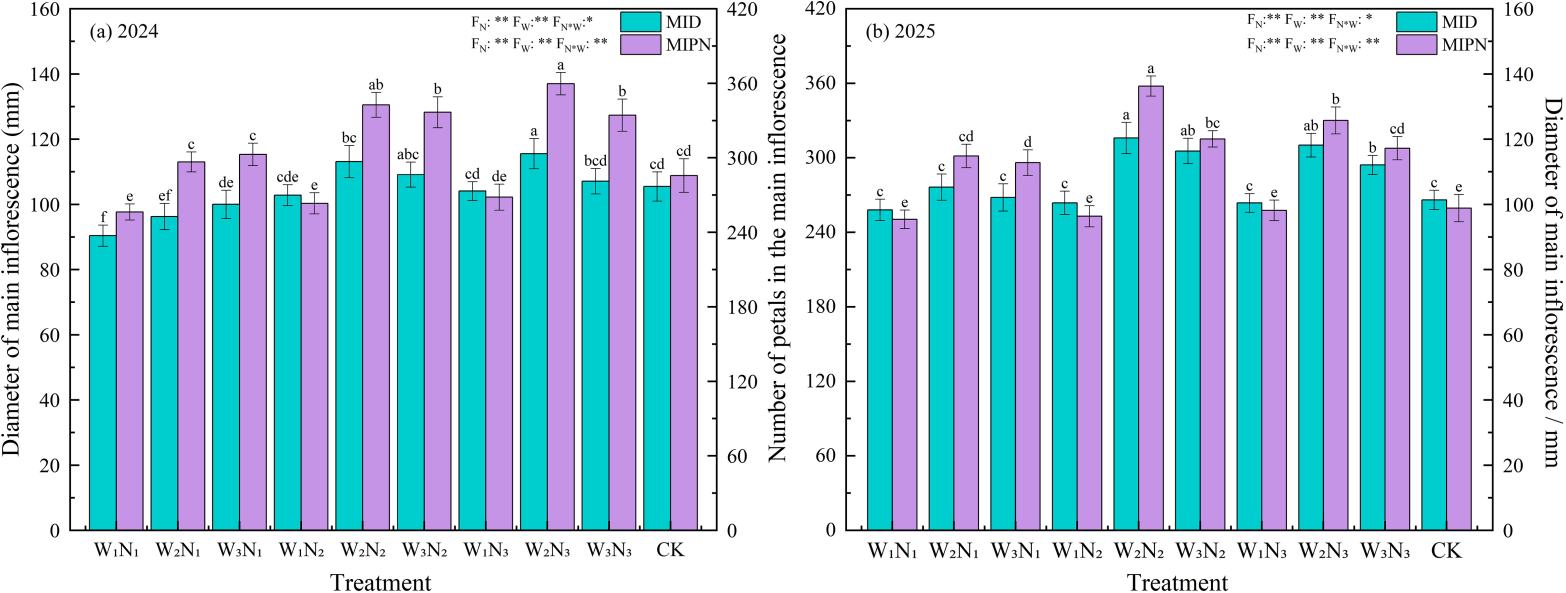
The impact of different irrigation methods and N application schemes on the diameter of the main inflorescence and petal number of Zinnia in 2024 **(a)** and 2025 **(b)**. MID represents the diameter of main inflorescence per plant, MIPN represents the number of main petals per plant. Different lowercase letters indicate significant differences between treatments at the *P* < 0.05 level. The error bar indicates the standard deviation. * and ** indicate significant differences at the *P* < 0.05 and P< 0.01 levels, respectively, and ns indicates no significant difference (*P* > 0.05).

Flower diameter and petal number are key indicators for evaluating the aesthetic value of Zinnias. Different combinations of water and N treatments exert significant regulatory effects on both. The results of two-factor variance analysis showed that the main effects of irrigation level (W) and N application level (N) on petal number reached a highly significant level (*P<* 0.01), and the interaction effect of water and N (W×N) on petal number was significant (*P* < 0.05). Flower diameter also exhibited significant differences among different water and N treatments (*P* < 0.05), clarifying the important regulatory role of synergistic water and N effects on the floral ornamental traits of Zinnias (**Figure 8**).

Two-year experimental data from 2024 to 2025 showed that the variation range of MID for each treatment was 90.40–120.36 mm, and the MIPN was 256.33 to 383.33. Among them, the two indicators of W2N2 and W3N3 treatments were significantly superior to other combinations, ranking as the optimal treatments. The MID of W2N2 treatment reached 117.96 mm, and the MIPN was 371.50. The MID of W3N2 treatment was 115.64 mm, and the MIPN was 348.17. Compared with CK, the MID of the above treatments was significantly increased by 11.71% to 13.45%, and the MIPN was significantly (*P* < 0.05) increased by 19.05% to 24.14%. In addition, compared with other water-N combinations, the increases in MID and MIPN were 4.47% to 20.01% and 6.67% to 20.01%, respectively.

Further analysis of trait responses under different N levels showed that at the N1 level, MID and MIPN of the W1N1, W2N1, and W3N1 treatments were all at low levels, indicating that low N supply had obvious limitations on the formation of flower traits. Under the N2 level, the MID (117.96 mm) and MIPN (371.50) of W2 treatment reached the peak, which were significantly (*P<* 0.05) higher than those of W1 and W3 treatment under the same N level. At the N3 level, the ornamental effect of W2 was the best, and its MID and MIPN values were significantly (*P<* 0.05) higher than those of the irrigation levels of W1 and W3. 

#### 3.4.2 Cut flower yield

**Figure 9:**
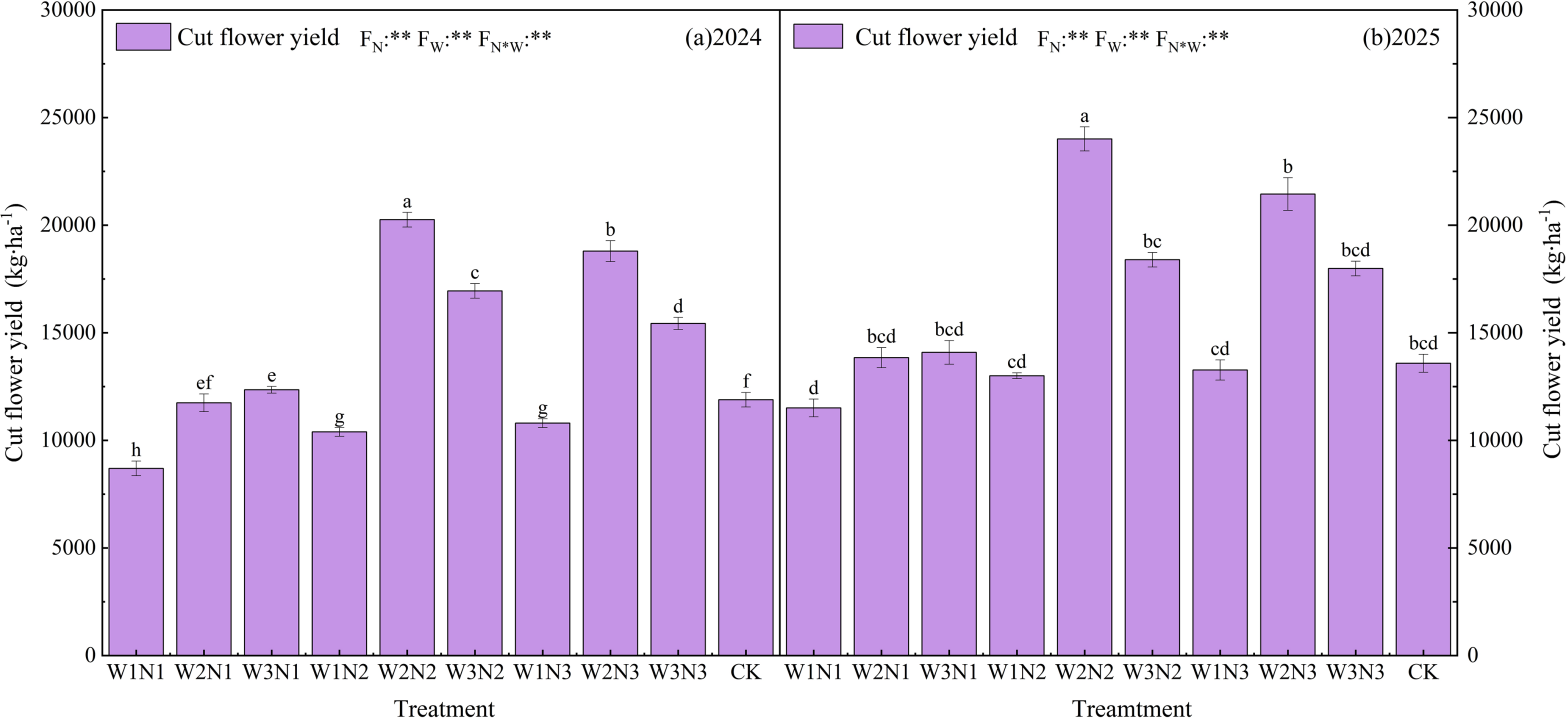
The impact of different irrigation methods and N application schemes on the cut flower yield of Zinnia in 2024 **(a)** and 2025 **(b)**. Different lowercase letters indicate significant differences between treatments at the *P* < 0.05 level. The error bar indicates the standard deviation. * and ** indicate significant differences at the *P* < 0.05 and P< 0.01 levels, respectively, and ns indicates no significant difference (*P* > 0.05).

Cut flower yield serves as the core indicator determining the commercial value of Zinnia, directly linking to growers’ economic benefits and market competitiveness. Its formation is significantly regulated by the synergistic interaction of water and nitrogen resources. As shown in **Figure 9** and supplementary data tables, extremely significant (*P* < 0.01) differences were observed in Zinnia cut flower yields under varying irrigation (W) and nitrogen application (N) treatments during 2024∼2025. Analysis of variance (ANOVA) results revealed that irrigation level (FW), nitrogen application level (FN), and their interaction (FN×W) all exerted extremely significant (*P* < 0.01) effects on cut flower yield, confirming that synergistic water-nitrogen regulation is a critical strategy for optimizing Zinnia cut flower yield.

The yield of Zinnia cut flowers in 2024 ranged from 8695.17 to 20254.30 kg·ha^-2^, with significant differences among treatments. The W2N2 treatment had the highest yield (20254.30 kg·ha^-2^), followed by W2N3 (18792.55 kg·ha^-2^), which were significantly (*P* < 0.05) higher than the control by 69.9% and 58.1% respectively. Under fixed nitrogen levels, the N1 level had a relatively low yield, significantly lower than N2 and N3 levels; under N2 and N3 levels, the W2 treatment was the best. Under fixed irrigation levels, the W2 treatment had a significantly higher yield, indicating that appropriate irrigation could increase the yield. The yield of Zinnia cut flowers in 2025 ranged from 8695.17 to 20254.30 kg·ha^-2^, which was overall higher than that in 2024, with a consistent response pattern. The W2N2 treatment remained the best (24008.69 kg·ha^-2^), followed by W2N3 (21,446.83 kg·ha^-2^), which were significantly (*P* < 0.05) higher than the control by 76.8% and 57.9% respectively. The yield at the N1 level was still relatively low, and under N2 and N3 levels, the W2 treatment was the best. The yield advantage of the W2 treatment was prominent, and excessive irrigation (W3) did not promote yield increase but might affect root vitality.

### 3.5 Photosynthetic physiological indices

#### 3.5.1 Trends of Pn, Tr, Gs, and Gi

**Figure 10:**
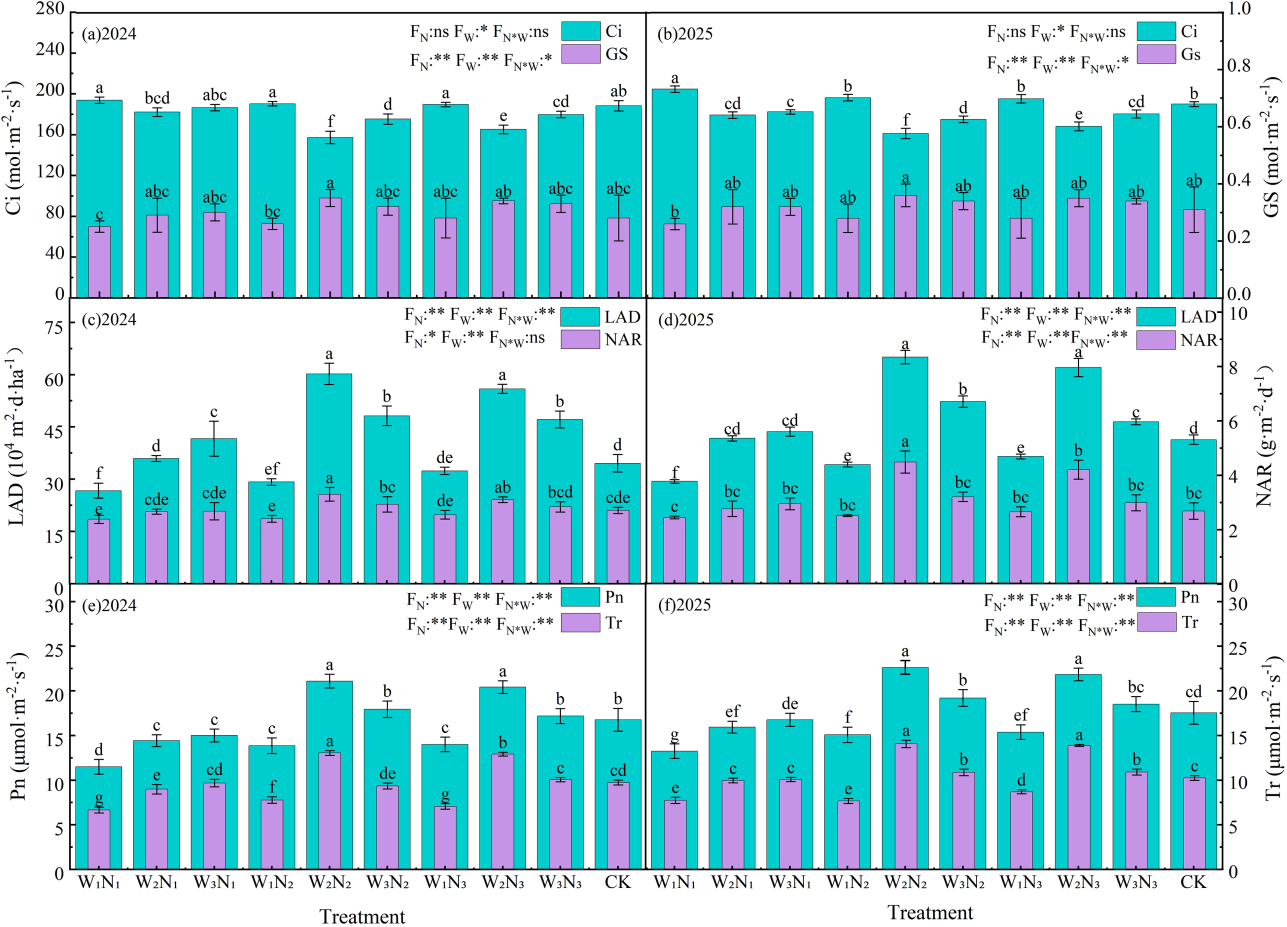
Effects of different irrigation methods and N fertilizer application schemes on photosynthetic physiological indicators of Zinnia in 2024 (a, c, e) and 2025 (b, d, f). Gi represents the intercellular CO_2_ concentration, Gs represents the stomatal conductance, LAD represents the leaf area duration, NAR represents the net assimilation rate, Pn represents the net photosynthetic rate, Tr represents the transpiration rate. Different lowercase letters indicate significant differences between treatments at the *P* < 0.05 level. The error bar indicates the standard deviation. * and ** indicate significant differences at the *P* < 0.05 and *P* < 0.01 levels, respectively, and ns indicates no significant difference (*P* > 0.05).

As shown in **Figures 10a, b, e, f**, under two different years and various water and nitrogen regulation levels, there were significant differences (P< 0.05) in Pn, Tr, Gs and Ci of Zinnia in terms of irrigation level (W) and nitrogen application rate (N). However, the interaction effect of W×N on Pn, Tr, Gs and Ci was not significant (P > 0.05). During the two years, the net photosynthetic rate (Pn) of Zinnia leaves reached the highest values under W2N2, W3N2 and W3N3 treatments, ranging from 17.95 μmol·m^-2^·s^-1^ to 21.10 μmol·m^-2^·s^-1^, which were significantly higher than those of other treatments by 14.36%∼83.48%, 12.11%∼77.57% and 4.42%~56.09%, respectively. Among them, the peak value of Pn occurred under the W2N2 treatment, which was 21.10 μmol·m^-2^·s^-1^ and 20.31μmol·m^-2^·s^-1^ in 2024 and 2025, respectively. During 2024 and 2025, compared with the control (CK), the Gs and Tr of W2N2 and W2N3 treatments were significantly increased by 28.97% and 17.17%, 35.69% and 34.14% on average, respectively. Gs reached the highest values of 0.39 mol·m^-2^·s^-1^ and 0.37 mol·m^-2^·s^-1^ under the above treatments, and Tr also rose to the peak levels of 14.0 μmol·m^-2^·s^-1^ and 13.5 μmol·m^-2^·s^-1^. Additionally, the highest values of intercellular CO_2_ concentration (Ci) in both years occurred under the W1N1 treatment, reaching relatively high levels of 193.86 mol·m^-2^·s^-1^ and 204.91 mol·m^-2^·s^-1^, which were 14.36% to 54.68% and 12.11% to 51.64% higher than those of other treatments. Under the condition of constant nitrogen application rate, as the degree of water stress increased, the Pn, Gs and Tr of Zinnia leaves all showed a downward trend, while Ci correspondingly increased. Under the same water conditions, as the nitrogen application rate increased, Pn, Gs and Tr all showed a trend of first increasing and then decreasing, while Ci showed the opposite response pattern. The above results indicate that the correlation characteristics among Gs, Ci, Pn and Tr are significantly regulated by the water-nitrogen coupling effect. Under an optimal water and nitrogen supply ratio (e.g., W2N2 and W2N3), the enhanced Gs effectively established a favorable physiological coordination among adequate CO_2_ supply, improved photosynthetic efficiency, and balanced water metabolism.

#### 3.5.2 LAD and NAR

As shown in **Figures 10c, d**, there were significant (*P* < 0.05) differences in LAD and NAR of Zinnia during the two years under different water and nitrogen management levels. Compared with W1, the LAD of W2 and W3 treatments increased significantly (*P* < 0.05) by 72.24% and 55.06% respectively in 2024, and by 68.76% and 42.32% respectively in 2025. Meanwhile, NAR also increased with the increase of photosynthetic potential. Compared with W1, the NAR of W2 and W3 treatments increased significantly (*P* < 0.05) by 23.99% and 15.37% respectively in 2024, and by 50.39% and 19.93% respectively in 2025. Under the condition of consistent irrigation levels, both LAD and NAR showed an initial increase followed by a stabilization trend with the increase of nitrogen fertilizer application. During the two growth seasons, compared with N4 treatment, the photosynthetic potential and net assimilation rate of N2 increased by an average of about 27.43%, which was significant; compared with N1 treatment, the average increase was about 32.33%, also reaching a significant (*P* > 0.05) level; however, there was no significant difference between N3 and N2 treatments. Additionally, in 2024 and 2025, compared with CK, the LAD and NAR of W2N2 treatment increased significantly by an average of 65.78% and 44.59% respectively. The above results indicate that different water and nitrogen management measures have significant effects on the photosynthetic characteristics and net assimilation capacity of crops.

### 3.6 Optimization of water and N supply patterns

#### 3.6.1 Determination of index weights based on entropy weight method

In this study, the EWM is used to determine the weights of decision indicators under decision factors such as selected Zinnia soil, growth, photosynthetic, physiological and biochemical indicators, and ornamental value. First, the original data were standardized to eliminate the influence of different index dimensions and make the data comparable. Then, the entropy value of each index was calculated. The entropy value reflects the degree of dispersion of the index. The greater the degree of dispersion, the smaller the entropy value, the more information the index provides, and the greater its weight accordingly. Finally, the weight of each index was calculated based on the entropy value, to provide a scientific basis for the subsequent comprehensive evaluation.

The standardized matrix X for the evaluation indicators:


$$X=\;\left[\begin{array}{cccccccccc} 0.0001 & 0.0001 & 0.0001 & 0.0001 & 0.0001 & 0.0001 & 0.0001 & 0.0001 & 0.0001 & 0.0001\\ 0.1964 & 0.2729 & 0.3338 & 0.3106 & 0.3586 & 0.5915 & 0.1312 & 0.3159 & 0.2240 & 0.2851\\ 0.3224 & 0.2864 & 0.4046 & 0.3767 & 0.4210 & 0.6425 & 0.1922 & 0.4317 & 0.2592 & 0.2977\\ 0.3356 & 0.0962 & 0.0843 & 0.2427 & 0.0827 & 0.2897 & 0.0804 & 0.0738 & 0.1327 & 0.3244\\ 1.0001 & 1.0001 & 1.0001 & 1.0001 & 1.0001 & 1.0001 & 1.0001 & 1.0001 & 1.0001 & 0.9950\\ 0.6655 & 0.5577 & 0.6215 & 0.7522 & 0.4588 & 0.8038 & 0.3904 & 0.5475 & 0.6292 & 0.7723\\ 0.5294 & 0.1318 & 0.1428 & 0.2683 & 0.1071 & 0.3096 & 0.0919 & 0.1369 & 0.1608 & 0.3515\\ 1.0001 & 0.7633 & 0.8089 & 0.9357 & 0.9357 & 0.9621 & 0.7593 & 0.6738 & 0.8329 & 1.0001\\ 0.7619 & 0.4887 & 0.4576 & 0.6151 & 0.5162 & 0.6921 & 0.2456 & 0.6212 & 0.5494 & 0.7225\\ 0.5209 & 0.1806 & 0.2521 & 0.4995 & 0.4399 & 0.4423 & 0.0899 & 0.2633 & 0.2190 & 0.4029 \end{array}\right]$$

The entropy weight method was used to calculate the index weight, and the calculation results are shown in **Table 3**.

**Table 3:** Weights calculation results in comprehensive evaluation indexes by entropy weight method.

Objective of the decision	Policy factors	Evaluating indicator	IEV	IUV	W (%)
Comprehensive evaluation of water and N supply patterns in Zinnias	Soil	SOM	0.9090	0.0910	7.6520
	Grow	LAI	0.8527	0.1473	12.3827
		DMA	0.8667	0.1333	11.2118
	Photosynthetic	Pn	0.9032	0.0968	8.1419
		Tr	0.9107	0.0893	7.5106
	Character	Ant	0.9210	0.0790	6.6444
		SP	0.7865	0.2135	17.9529
	Ornamental value	NF	0.8692	0.1308	10.9993
		CFY	0.8895	0.1105	9.2869
		MID	0.9023	0.0977	8.2175

SOM, soil organic matter (g·kg^-1^); LAI, leaf area index (leaf area per unit area) (m^2^·plant^-1^); DMA, dry matter accumulation (per unit area) (g·plant^-1^); Pn and Tr, net photosynthetic rate and transpiration rate (μmol·m^-2^·s^-1^); Ant, anthocyanin (mg·g^-1^); SP, soluble protein (mg·g^-1^); NF, the number of flowers per plan (plant^-1^); CFY, cut flower yield (kg·ha^-2^); MID, diameter of the main inflorescence (mm); IEV represents the information entropy value e; IUV, the information entropy value d; and W, the weight occupied. Different lowercase letters indicate significant differences between treatments at the *P* < 0.05 level. * and ** indicate significant differences at the *P* < 0.05 and *P* < 0.01 levels, respectively, and ns indicates no significant difference (*P* > 0.05).

#### 3.6.2 Comprehensive evaluation and optimization of water and nitrogen supply modes

In this study, 10 treatments and 8 evaluation indicators including selected yield were used. Based on the entropy weighting method and the TOPSIS method, the comprehensive evaluation of the water and N supply mode of Zinnia was carried out, and the evaluation method adopted an objective weight calculation method to avoid the subjectivity of artificial weighting. Firstly, according to the information entropy values of each index calculated earlier, the entropy weights of each index are further calculated, which can accurately reflect the importance of each evaluation index in the comprehensive evaluation of Zinnia water and N supply mode. Then, the TOPSIS method was used to sort the 10 treatments according to the closeness of each treatment to the ideal solution. Through this method, the performance of each treatment under different evaluation indicators can be comprehensively and comprehensively considered, to select the water and N supply mode of Zinnia with the best comprehensive performance in biomass, photosynthetic characteristics, leaf characteristics, ornamental value and other aspects, so as to provide a scientific basis for the efficient planting of Zinnias.

A weighted matrix was constructed as follows:


$$\text{R}=\left[\begin{array}{cccccccccc} 0.0000 & 0.0000 & 0.0000 & 0.0000 & 0.0000 & 0.0000 & 0.0000 & 0.0000 & 0.0000 & 0.0000\\ 0.0151 & 0.0317 & 0.0356 & 0.0237 & 0.0382 & 0.0369 & 0.0221 & 0.0326 & 0.0251 & 0.0220\\ 0.0233 & 0.0333 & 0.0426 & 0.0288 & 0.0449 & 0.0401 & 0.0324 & 0.0446 & 0.0291 & 0.0230\\ 0.0242 & 0.0112 & 0.0089 & 0.0185 & 0.0088 & 0.0181 & 0.0135 & 0.0076 & 0.0149 & 0.0250\\ 0.0681 & 0.1162 & 0.1052 & 0.0764 & 0.1067 & 0.0624 & 0.1685 & 0.1032 & 0.1121 & 0.0767\\ 0.0481 & 0.0648 & 0.0654 & 0.0575 & 0.0489 & 0.0501 & 0.0658 & 0.0565 & 0.0706 & 0.0596\\ 0.0382 & 0.0153 & 0.0150 & 0.0205 & 0.0114 & 0.0193 & 0.0155 & 0.0141 & 0.0180 & 0.0271\\ 0.0722 & 0.0887 & 0.0851 & 0.0715 & 0.1041 & 0.0600 & 0.1279 & 0.0695 & 0.0934 & 0.0771\\ 0.0550 & 0.0568 & 0.0481 & 0.0470 & 0.0551 & 0.0432 & 0.0414 & 0.0641 & 0.0616 & 0.0557\\ 0.0376 & 0.0210 & 0.0265 & 0.0382 & 0.0469 & 0.0276 & 0.0150 & 0.0272 & 0.0246 & 0.0331 \end{array}\right]$$

Comprehensive evaluation calculation is performed using the entropy weight-TOPSIS method, and the calculation results were shown in **Table 4**.

**Table 4:** Comprehensive evaluation results of Zinnia water and N supply modes based on entropy weight TOPSIS method.

Treatment	$D_{i}^{+}$	$D_{i}^{-}$	C_i_	Sort
W1N1	0.329	0.035	0.096	10
W2N1	0.246	0.092	0.273	6
W3N1	0.227	0.111	0.328	5
W1N2	0.288	0.051	0.151	9
W2N2	0.004	0.328	0.988	1
W3N2	0.153	0.187	0.550	3
W1N3	0.276	0.066	0.193	8
W2N3	0.066	0.275	0.807	2
W3N3	0.178	0.168	0.486	4
CK	0.249	0.097	0.282	7

$D_{i}^{+}$/ $D_{i}^{-}$, the distance between the evaluation object and the positive/negative ideal. C_i_, the evaluation score.

According to the Ci calculation principle, a larger $D_{i}^{+}$ value leads to a larger Ci value and better processing synthesis performance. **Table 4** shows that each index of W2N2 is balanced and the Di^−^ value is high, which demonstrates significant advantages in compensating for disadvantages. This is due to the improvement of resource utilization efficiency through a reasonable water-to-N ratio, resulting in an optimal comprehensive evaluation. The overall performance of W2N3 was good, but there was still room for improvement in specific indicators, which could be further optimized by fine-tuning the details of water and N supply. The Ci values of CK control and W1N1 treatment were lower, indicating that the water and N supply model was insufficient. The growth potential of CK was limited due to a lack of reasonable nutrient supply. Due to the imbalance in the water-to-N ratio, the nutrient supply for W1N1 was insufficient, which reduced absorption and utilization efficiency; thus, the growth performance of both W1N1 treatments was unsatisfactory. In practical production, priority should be given to implementing the W2N2 water-N ratio scheme while considering both Zinnia growth demand and soil conditions to balance adequate nutrient supply with efficient resource utilization. For W2N3, it is necessary to improve short-term indicators and fine-tune either time or frequency or concentration regarding water and N supply. For CK and W1N1 treatments, specific reasons for nutrient deficiency or imbalance should be clarified; additionally, optimizing growth environments by increasing nutrient application while adjusting ratios between water and N will enhance growth quality as well as biomass.

## 4 Discussion

### 4.1 Soil enzyme activity and SOM

Water and N regulation, as a key management practice in agricultural production, has a significant impact on soil fertility supply, involving multiple aspects such as soil organic matter and nutrient cycling (Ma et al., 2024b; Wu et al., 2019). Especially in arid regions, where water resources are increasingly scarce, improper soil-water management and low fertilizer use efficiency pose severe challenges to agricultural production. Numerous studies have demonstrated that blindly increasing irrigation and fertilization rates fails to yield a sustained positive effect on soil fertility. Conversely, excessive water and nutrient inputs may disrupt soil structure, reduce (Ma et al., 2024a; Wu et al., 2019) microbial activity, and inhibit soil enzyme activity (Luo et al., 2024; Shen et al., 2025; Zhou et al., 2024). On the contrary, scientific water and fertilizer management can significantly enhance soil enzyme activity, promote the decomposition of organic matter and nutrient cycling, and thereby improve soil fertility conditions (Korkanç, 2024; Shouguo et al., 2023). In addition, scientific water and fertilizer management can also reduce nutrient loss and increase fertilizer utilization efficiency, thereby ensuring crop yields while maintaining the health and stability of the soil ecosystem (Wanting et al., 2022; Xing et al., 2024). In our research, moderate water and nitrogen regulation usually promotes the decomposition and transformation of organic matter in the soil, enhancing the availability and supply capacity of soil nutrients. For instance, under a constant nitrogen application level, as the amount of irrigation and nitrogen fertilizer increases further, it is observed that soil enzyme activities (urease, catalase, sucrase) and organic matter content initially rise and then decline with the increase in irrigation and nitrogen fertilizer application. Meanwhile, excessive nitrogen application increases the risk of soil degradation, inhibits soil enzyme activities, and leads to a decreasing trend in organic matter content. These results indicate that in agricultural production, excessive application of nitrogen fertilizer and irrigation not only fail to sustainably enhance soil fertility but may also cause resource waste and environmental pollution, exerting adverse effects on the health of the soil ecosystem. Our research findings are consistent with previous studies, indicating that an appropriate combination of irrigation and nitrogen fertilizer is more beneficial for optimizing the physical and chemical properties of soil than excessive application (Ji et al., 2017; Li et al., 2019; Wang et al., 2023a; Yi et al., 2022). For example, Yi et al. (2022) found that the addition of different proportions of exogenous N in various forms promoted the growth of Medicago sativa by increasing the activities of redox enzymes such as hydroxylamine reductase, catalase, and polyphenol oxidase. Ji et al. (2017) also pointed out that N addition can synergistically stimulate soil glycosidase activity and soil respiration, indicating that N promotes soil microbial-mediated carbon cycling. In addition, another study found that short-term drought stress can disturb soil microbial community composition and functional gene expression, thereby significantly affecting the differences in soil organic carbon fractions and enzyme activity levels (Wang et al., 2023a). It can be inferred from this that water and nitrogen regulation play a core role in the regulation process of soil enzyme activity, SOM microbial community structure and their activity. Optimizing water and N regulation models can effectively alleviate water scarcity and improve agricultural productivity and product quality.

### 4.2 Physiological and biochemical indicators

Physiological and biochemical indicators are key carriers reflecting the adaptive strategies of plants to environmental changes and the level of resource supply (Gao et al., 2024; Su et al., 2024). Especially in arid and semi-arid regions, the fluctuations in water and nitrogen supply have a significant impact on the physiological and biochemical processes of plants (Ma et al., 2025). In these regions, plants maintain cell turgor pressure and normal metabolic functions by adjusting the contents of osmoregulatory substances such as proline and soluble sugars (Minguo et al., 2021). Meanwhile, the form and supply level of nitrogen directly affects the activity of enzymes related to nitrogen metabolism in plants, thereby regulating the synthesis rate of amino acids and proteins (Long et al., 2026). In addition, plants also respond to the oxidative damage caused by water stress by altering the activity of the antioxidant enzyme system. This adaptive regulation plays a crucial role in maintaining the stability of plant growth (Shukla et al., 2025). This study revealed that under drought conditions, water and nitrogen regulation exerted a significant (*P<* 0.05) effect on the physiological and biochemical indices. With increasing irrigation volume, the contents of anthocyanins, lutein, soluble proteins, and soluble sugars in 1innia all exhibited an upward trend, and its appearance quality was correspondingly improved due to the enhancement of physiological and biochemical indices. However, no significant difference was observed between the mild water deficit (W2) and full irrigation (W3) treatments. This conclusion is largely consistent with existing research findings. For instance, studies have demonstrated that as the intensity of drought stress increases (from 200 mL of water in mild drought to 25 mL in extreme drought), the growth of Begonia semperflorens is significantly inhibited, accompanied by a decline in its ornamental value and physiological properties (Zhao et al., 2024). Additionally, Long et al. (2004) research on petunia pointed out that applying 200 mg of N per kilogram of soil is most suitable for flower growth and ornamental quality; meanwhile, they found that different types of slow-release fertilizers under the same N amount also have significant effects on the growth and ornamental quality of petunia. This result aligns with the phenomenon observed in this study where the contents of anthocyanins, lutein, soluble proteins, and soluble sugars in Zinnia peaked at the medium nitrogen level (N2). The study further found that with the increase in nitrogen fertilizer application, the accumulation of the nutritional components in Zinnia showed a trend of first increasing and then decreasing. Therefore, in arid or semi-arid regions, precisely regulating soil moisture to optimize the nutrient absorption efficiency of plants, thereby enhancing their growth performance and quality, holds important implications for the cultivation and management of ornamental plants.

### 4.3 Ornamental value

The ornamental value is the core criterion for evaluating ornamental cut flowers. It integrates visual features such as the shape, color and yield of the flowers, which are strictly regulated by the level of resource supply (Vaghefi et al., 2026). In the cultivation of ornamental plants, adequate water supply and appropriate nitrogen fertilizer application can significantly enhance the fullness of flower shapes and the vividness of their colors, making the flowers more attractive (Kitty et al., 2019). At the same time, good water and nitrogen management can also increase the number of flowers of ornamental plants, raise the yield per unit area, and thereby further enhance their ornamental value (Wilkinson et al., 2019). Conversely, drought stress or insufficient nitrogen supply can lead to small and withered flower shapes, dull colors, and even affect the number of flowers and the flowering period, significantly reducing the ornamental value of the plants (Toscano et al., 2017). Studies have shown that under appropriate irrigation conditions, combined with N application levels of N2 and N3, the plant height, stem diameter, leaf area, number of lateral branches, and number of flowers of Zinnia significantly increased, resulting in optimal ornamental performance. He et al. (2023) found that for ornamental plants, appropriate N application can effectively promote the growth of branches and leaves, increase plant height and crown width, thereby significantly enhancing their ornamental value. Cardarelli et al. (2015) indicated that optimizing nitrate N application rate can improve its growth index, quality, and mineral composition. Additionally, research has indicated that the combination of micro-sprinkler irrigation technology and rain-shelter cultivation can significantly increase root yield and quality of Panax notoginseng, reduce the incidence of root rot, and improve water use efficiency (Zang et al., 2024). All these conclusions are like those obtained from this experiment. In conclusion, precisely regulating water and nitrogen supply not only benefits the healthy growth of plants and the optimization of ornamental traits, but also reduces nutrient loss and environmental pollution, promoting the greening and sustainable development of ornamental plant production.

### 4.4 Physiological metabolic characteristics

Water and N regulation exerts profound impacts on the physiological processes of ornamental plants, such as photosynthesis, N metabolism, and water use efficiency (Deng et al., 2025; Guan et al., 2023). N supply directly affects plant chlorophyll synthesis and photosynthetic efficiency (Hui et al., 2022). Water status is equally crucial for photosynthesis; drought stress limits plant growth by influencing stomatal conductance, photosynthetic rate, and water use efficiency (Li et al., 2020). Therefore, appropriate water and N supply is a key factor in maintaining the normal growth and development of ornamental plants, improving photosynthetic efficiency, and enhancing ornamental value (Cechin and de Fátima Fumis, 2004). Our research found that under appropriate water and nitrogen regulation conditions, the Pn, Tr and Gs of Zinnia were significantly (*P* < 0.05) higher than those of CK. With the improvement of photosynthetic capacity, Ci decreased accordingly, which effectively promoted the increase of LAD and NAR, and was conducive to the synthesis and accumulation of carbohydrates. Yan et al. (2024) research on quinoa also showed that different N fertilizer doses had significant effects on the phenology, photosynthetic fluorescence parameters, and yield of quinoa. During different growth stages of quinoa, the SPAD value varied with N levels, usually reaching higher values at the N2 or N3 level, with distinct response patterns across growth stages. Meanwhile, Wang et al. (2020) conducted a study on isatis root in the arid oasis of central Hexi Corridor, finding that mild water deficit had no significant impact on the leaf photosynthetic capacity (indicated by Pn, Tr, and Gs) of isatis root. Moreover, there was a certain compensatory response after rewatering, which could improve yield and irrigation efficiency. These studies all indicate that water and N regulation play an indispensable role in the photosynthetic and physiological processes of plants. In addition to its effects on photosynthesis, appropriate water and N conditions can optimize plant N metabolism (Nie et al., 2024). Under reasonable water and N supply, the efficiency of N uptake, assimilation, and reuse in plants is improved. This facilitates the synthesis of more N-containing compounds such as proteins and nucleic acids in plants, thereby promoting plant growth and development (Kang et al., 2023). Furthermore, from the perspective of water use efficiency, an appropriate water-N combination enables plants to maintain a high level of physiological activity while consuming less water (Kamran et al., 2022; Yamori et al., 2020). Therefore, an appropriate combination of water and nitrogen can enable plants to maintain a high level of physiological activity while consuming less water. This is particularly important for ornamental plants growing in arid or semi-arid regions, as it not only saves water resources but also ensures that the plants have good ornamental traits and economic value.

### 4.5 Cut flower yield

The cut flower yield (CFY), as a core indicator reflecting the production potential of ornamental plants, responds to water and nitrogen regulation closely related to species characteristics, regional ecological conditions, and cultivation management measures (Kamran et al., 2022). In the practice of horticultural production, the coordinated regulation of water and nitrogen has a significant impact on the yield of ornamental plant cut flowers. Appropriate water supply and optimized nitrogen application can effectively promote the vegetative growth and reproductive development of plants, significantly increase the number of flowers per plant, flower diameter and the number of flower branches, thereby enhancing the yield of cut flowers per unit area (Bar-Yosef et al., 2009; Porto et al., 2014). This study focused on the production of Zinnia cut flowers in the arid oasis area of the Hexi Corridor, systematically exploring the effects of water and nitrogen coupling regulation on yield formation. The results showed that under the optimal water and nitrogen combination treatment, the cut flower yield reached its peak in both 2024 and 2025, significantly higher than the yields of similar varieties and various mainstream ornamental cut flowers reported by previous studies (Kumar and Sudip, 2022; R et al., 2025), strongly confirming the scientific nature and practical effectiveness of the water and nitrogen coordinated management strategy suitable for this region. The research by Paul Ngubeni et al (Ngubeni and Wahome, 2022). found that under greenhouse conditions, different irrigation regimes (water volume and irrigation intervals) had significant effects on the yield and quality parameters of carnations (*Dianthus caryophyllus L.*). Yusuf Ucar et al. (2017) found that different irrigation amounts and nitrogen fertilizer dosages would affect the flower and essential oil yields as well as water use efficiency of Rosa. The above conclusion is basically consistent with the results of this study, further confirming the key role of water and nitrogen co-regulation in the production of cut flowers of ornamental plants.

## 5 Conclusions

This study systematically explored the effects of different water and nitrogen combinations on the growth and development of Zinnia, the ornamental quality of cut flowers, and the nutrient environment of rhizosphere soil, and identified the optimal water and nitrogen management thresholds for achieving high-quality and efficient cultivation. The results showed that in two consecutive growing seasons, a mild water deficit combined with a moderate nitrogen application rate could significantly enhance key ornamental traits such as plant height, stem diameter, flower diameter, number of flower branches, and vase life, while maintaining high photosynthetic efficiency, antioxidant enzyme activity, and nitrogen use efficiency, demonstrating good physiological coordination. Further analysis revealed that the synergistic effect of mild water deficit and medium to high nitrogen levels could significantly promote the accumulation of secondary metabolites such as anthocyanins and lutein in petals, as well as osmotic adjustment substances such as soluble sugars and soluble proteins, thereby enhancing the drought resistance and postharvest quality stability of the plants. Notably, although moderate increases in nitrogen fertilizer and irrigation could help increase the total nitrogen and organic matter content in the 0~60 cm soil layer, excessive input could lead to soil nutrient loss, C/N ratio imbalance, and shifts in microbial community structure. Long-term implementation would weaken soil health functions and restrict the sustainable production of Zinnia. Based on entropy weight method-based weighting and TOPSIS comprehensive evaluation, the W2N2 treatment exhibits the highest closeness coefficient (0.988) and is therefore determined as the optimal collaborative management mode that balances soil ecological health, plant physiological robustness, and the commercial quality of cut flowers. Although this study focused on Zinnia, the revealed water-nitrogen interaction laws and multi-objective optimization paths have good reference significance for other annual cut flower crops with similar light-loving and drought-tolerant characteristics (such as marigold and calendula). Future research can be extended to different ecological types of flower species, combined with long-term positioning experiments and multi-omics analysis, to systematically evaluate the interspecific applicability, regional adaptability, and climate response elasticity of water-nitrogen combinations strategies, thereby providing more universal theoretical support and technical paradigms for the green, precise, and sustainable development of the characteristic flower industry in arid oasis areas of China.

## Data Availability

The original contributions presented in the study are included in the article/supplementary material. Further inquiries can be directed to the corresponding author.
